# YYFZBJS ameliorates colorectal cancer progression in *Apc*^*Min/+*^ mice by remodeling gut microbiota and inhibiting regulatory T-cell generation

**DOI:** 10.1186/s12964-020-00596-9

**Published:** 2020-07-16

**Authors:** Hua Sui, Lu Zhang, Kaijuan Gu, Ni Chai, Qing Ji, Lihong Zhou, Yan Wang, Junze Ren, Limei Yang, Bimeng Zhang, Jing Hu, Qi Li

**Affiliations:** 1 Department of Medical Oncology, Shuguang Hospital, Shanghai University of Traditional Chinese Medicine, 528 Zhangheng Rd, Shanghai, 201203 P.R. China; 2 Preclinical Medicine College of Shanghai University of Traditional Chinese Medicine, 1200 Cailun Rd, Shanghai, 201203 P.R. China; 3 Yueyang Hospital of Integrated of Traditional Chinese and Western Medicine, Shanghai University of Traditional Chinese Medicine, Shanghai, 200437 P.R. China; 4 Changhai Hospital of Traditional Chinese Medicine, Naval Medical University, Shanghai, 200433 P.R. China; 5 Department of Acupuncture and Moxibustion, Shanghai General Hospital, Shanghai Jiao Tong University School of Medicine, No. 100 Haining Rd, Hongkou District, Shanghai, 200080 P.R. China; 6 Academy of Integrative Medicine, Shanghai University of Traditional Chinese Medicine, Shanghai, 201203 P.R. China

**Keywords:** Colorectal Cancer, *Apc*^*Min/+*^ mice, Gut microbiota, Fecal microbiota transplantation, Regulatory T cell, Immune, Traditional Chinese herb medicine

## Abstract

**Background:**

Progression of Colorectal cancer (CRC) is influenced by single or compounded environmental factors. Accumulating evidence shows that microbiota can influence the outcome of cancer immunotherapy. T cell, one of the main populations of effector immune cells in antitumor immunity, has been considered as a double-edged sword during the progression of CRC. Our previous studies indicate that traditional Chinese herbs (TCM) have potential anticancer effects in improving quality of life and therapeutic effect. However, little is known about the mechanism of TCM formula in cancer prevention.

**Methods:**

Here, we used C57BL/6 J *Apc*^*Min/+*^ mice, an animal model of human intestinal tumorigenesis, to investigate the gut bacterial diversity and their mechanisms of action in gastrointestinal adenomas, and to evaluate the effects of Yi-Yi-Fu-Zi-Bai-Jiang-San (YYFZBJS) on of colon carcinogenesis in vivo and in vitro. Through human-into-mice fecal microbiota transplantation (FMT) experiments from YYFZBJS volunteers or control donors, we were able to differentially modulate the tumor microbiome and affect tumor growth as well as tumor immune infiltration.

**Results:**

We report herein, YYFZBJS treatment blocked tumor initiation and progression in *Apc*^*Min/+*^ mice with less change of body weight and increased immune function. Moreover, diversity analysis of fecal samples demonstrated that YYFZBJS regulated animal’s natural gut flora, including *Bacteroides fragilis*, *Lachnospiraceae* and so on. Intestinal tumors from conventional and germ-free mice fed with stool from YYFZBJS volunteers had been decreased. Some inflammation’ expression also have been regulated by the gut microbiota mediated immune cells. Intestinal lymphatic, and mesenteric lymph nodes (MLN), accumulated CD4+ CD25+ Foxp3 positive Treg cells were reduced by YYFZBJS treatment in *Apc*^*Min/+*^ mice. Although YYFZBJS had no inhibition on CRC cell proliferation by itself, the altered Tregs mediated by YYFZBJS repressed CRC cancer cell growth, along with reduction of the phosphorylation of β-catenin.

**Conclusions:**

In conclusion, we demonstrated that gut microbiota and Treg were involved in CRC development and progression, and we propose YYFZBJS as a new potential drug option for the treatment of CRC.

Video abstract

**Graphical abstract:**

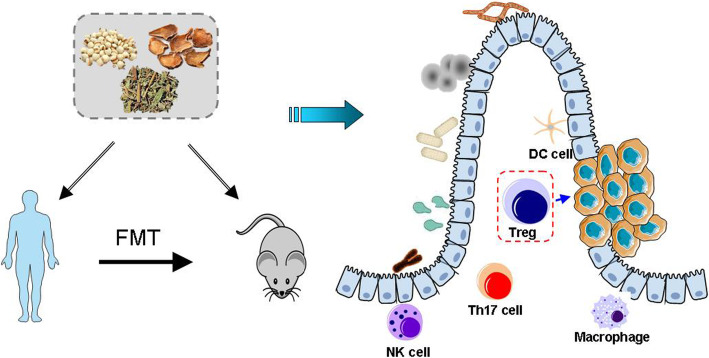

## Background

CRC is one of the most common cancers with an annual incidence of nearly 1 million cases worldwide and an annual mortality of more than 600,000 patients [1]. Accumulating evidence suggests that the gut microbiota, chronic inflammation, host genetic predisposition, and environmental factors have been linked with the progression of CRC [2]. Previous studies have identified several bacteria that can promote carcinogenesis by different mechanisms, such as Bacteroides, which can alter bile acid metabolism and/or increase IL-22 levels [3]; Fusobacterium nucleatum which can activate the autophagy pathway and alter colorectal cancer chemotherapeutic response through Toll-like receptor pathways [4] and Eschericia which can induce colonic infection in the bacterial mediated CRC [5]. Interestingly, the fecal samples of CRC patients can induce intestinal tumorigenesis and colon cell proliferation in colon tumour model mice, as well as increase the expression of inflammatory genes and carcinogenic factors [6]. Fecal microbiota transplantation (FMT) is one procedure that involves the complete restoration of the entire fecal microbiota instead of a single agent or combination of agents. Emerging studies have found significant differences in intestinal microbial communities between CRC patients and healthy individuals [7].

A key player involved in the processes of gut microbiota and tumorigenesis is the tumor-infiltrating immune cell, which is popular in the intestinal tract and contains a myriad of immune cells, such as macrophages, dendritic cells, neutrophils, and lymphocytes (T cells), start from naive T cells to undergo differentiation processes during which they acquire the capacity to produce distinct sets of effector cytokines [8]. Different lineages derived from CD4+ T cells including Th1, Th2, Th17, regulatory T, and Tr1 cells, have extensive effects in cancer development. Current studies have mainly explored the changes of the circulating levels of cytokines that reflect the balance of the four T cells, i.e. plasma levels of interferon gamma (IFN-γ), interleukin-6/10 (IL-6/10), and tumor necrosis factor-α (TNF-α)] [9, 10]. In recent years, clinical observations indicated that CD4+ CD25+ regulatory T cells (Tregs) played a promoting role in various cancers such as gastric, colorectal, pancreatic cancers and hepatocellular carcinoma [11–13]. Moreover, Tregs was reported to suppress immune responses and hinder suppression of tumor growth in preclinical models [14].

Emerging studies have highlighted a key role for the commensal microbiota in the immunoregulatory responses, probably through affecting T-helper (TH) and T regulatory cells (Tregs) [15]. For example, *L. reuteri* together with a tryptophan-rich diet can reprogram intraepithelial CD4+ T cells into immunoregulatory T cells [16]. Clostridia clusters IV and XIVa promote Treg differentiation [17, 18], and *Lactobacillus rhamnosus* [19] convert mucosal dendritic cells toward tolerogenic profiles via secreting IL-10 and TGF-β. Although gut microbiota has been identified as a trigger for mucosal Treg/Th17 balance and is sufficient to promote autoimmunity in murine models [20], no microbial promoter of Treg has yet been found to be associated with occurrence of human adenoma or colorectal adenocarcinoma (CRC). However, there is emerging data to link different bacteria, such as *Faecalibacterium prausnitzii* (F. prausnitzii), *Bifidobacterium longum* (B. longum), and *Bacteroides fragilis*, to their ability to induce T cell differentiation and cytokine production in the development of CRC [21, 22].

Development of CRC begins with the formation of aberrant crypt foci, which are the earliest recognized lesions [23]. At this stage, genetic alterations such as adenomatous polyposis coli (*Apc*) gene silencing may occur, which successively lead to adenomatous polyp formation. As other mutations accumulate, the tumor ultimately progresses to invasive adenocarcinoma. *Apc*^*Min/+*^ mice, a genetically engineered mouse model that has a mutation in the *Apc* gene, usually serve as a well-characterized animal model for human familial adenomatous polyposis [24]. Ki67 and Proliferating Cell Nuclear Antigen (PCNA) proteins are standard markers of cell proliferation, thus commonly used to help assess malignancy grades of cancer [25]. The *Apc*^*Min/+*^ mice are often used as a well-recognized spontaneous CRC model, highly expressing Ki67 and PCNA. Although studies highlighted the close involvement of Treg cells in CRC tumorigenesis in the *Apc*^*Min/+*^ mouse model [26], the underlying molecular mechanism remains largely enigmatic.

Yi-Yi-Fu-Zi-Bai-Jiang-San (YYFZBJS), a thousand-year-old prescription from the Golden Chamber, is commonly used in traditional Chinese medicine (TCM) to treat gastrointestinal disorders [27, 28]. It is composed of three herbs: Yi-yi-ren (Semen Coicis), Fu-Zi (monkshood), Bai-jiang-cao (Herba Patriniae), which are in a ratio of 30:6:15. Recently, Semen Coicis, Herba Patriniae, and monkshood are found to have multiple pharmacological activities, including anti-cancer effect [28–30]. Notably, Yi-yi-ren and Bai-jiang-cao, the most abundant ingredients in the recipe, showed anti-proliferative efficacy in several human cancer cell lines, as well as a suppressive effect on the development of aberrant crypt force (ACF) in Azoxymethane (AOM) treated mice [31]. Our previous work demonstrated that some TCM inhibited the proliferation of CRC cells in vivo and in vitro [32, 33]. However, the anti-proliferation effect of YYFZBJS on the intestinal tumor is poorly understood.

In the current study, we investigated the effect of YYFZBJS in a spontaneous intestinal tumor model of *Apc*^*Min/*+^ mice. Gavaging germ-free *Apc*^*Min/*+^ mice with stool from healthy controls and YYFZBJS volunteers, we demonstrated that stool from YYFZBJS volunteers altered dysregulated inflammation and oncogenic pathways and inhibited intestinal tumorigenesis. We characterized the importance of Treg and expression levels of the related factors in spleen, MLN, LPL, and PBMC (Peripheral blood mononuclear cell) of the mice, in order to find the possible mechanisms involving in the anti-cancer action of TCM prescriptions, and the role of Treg cells in spontaneous intestinal carcinogenesis.

## Materials and methods

### Cell culture and reagents

Human colorectal adenocarcinoma HCT116 cell and Mice colorectal adenocarcinoma MC-38 cell were purchased from the Shanghai Cell Collection (Shanghai, China). They were cultured in RPMI 1640, which were all supplemented with 10% fetal bovine serum (Gibco, NY, USA), 2 mM glutamine, 100 units/ml streptomycin and penicillin (Invitrogen, Carlsbad, CA). The cells were grown at 37 °C in a humidified 5% CO_2_ atmosphere. Monoclonal antibodies specific for Ki67 (ab1667), PCNA (ab92552) and β-actin (ab179467) were obtained by Abcam plc., Cambridge, UK.

### Mouse strains and breeding

**Fig. 1:**
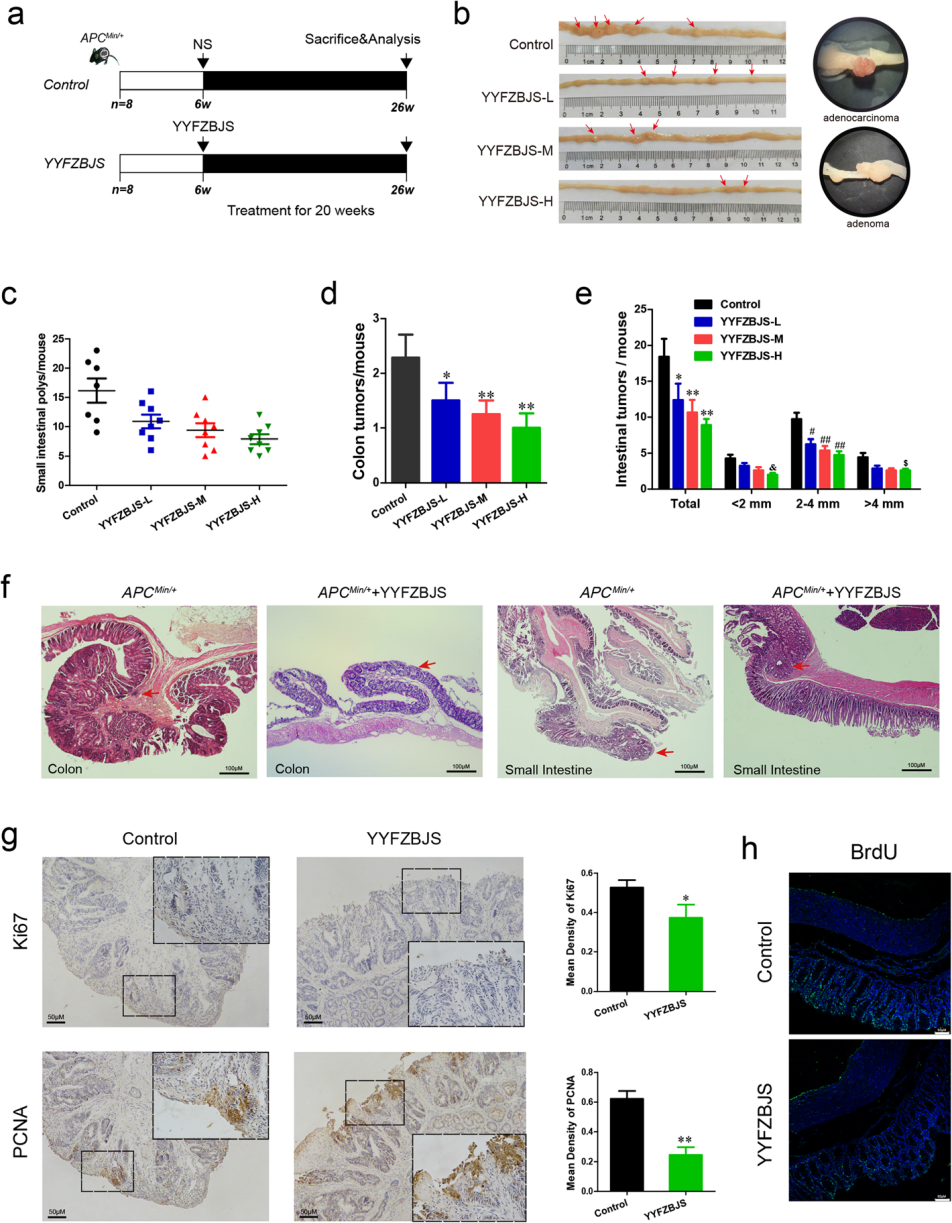
Experimental design and effect of YYFZBJS in intestinal tumorigenesis. **a** Experimental design indicating the timing of intragastric administration and organization of groups. **b** Macroscopic view of the representative mouse intestinal shows several polypoid and discoid colonic tumors from different groups of *Apc*^*Min/+*^ mice after treatment with YYFZBJS for 20 weeks. **c** The number of intestinal polyps in small intestinal from different groups of *Apc*^*Min/+*^ mice after treatment with YYFZBJS for 20 weeks. **d** The number of intestinal polyps in the colon from different groups of *Apc*^*Min/+*^ mice after treatment with YYFZBJS for 20 weeks. The data are presented as the mean ± SD from at least three experiments. **e** The tumor size distribution in the intestine was listed and compared with control. **f** Left: typical adenomatous polyp seen in infected *Apc*^*Min/+*^ mice showing high-grade dysplasia and carcinoma in situ. Middle: adenomatous intestinal polyp with the early invasion of neoplastic glands into the muscular layers often seen in *Apc*^*Min/+*^ mice. Right: minute polyp with remnant dysplastic glands close to the surface epithelium. This typical regressive intestinal cancer morphology is seen throughout the intestine in mice. Red arrows indicated adenocarcinoma cell. Magnification bars, 100 μM. **g**&**h** Immunohistochemical staining with an antibody against PCNA, Ki67, BrdU in control group and YYFZBJS treatment group. Magnification bars, 50 μM. Data are given as means ± SD of 8 animals per experimental group, with Welch’s correction, one-tailed t-test. ^#^*P* < 0.05, ^##^*P* < 0.01; *P < 0.05, **P < 0.01, ^&^*P* < 0.05, ^$^*P* < 0.05 vs. control

*Apc*^*Min/+*^ mice on a C57BL/6 J background were originally obtained from the Jackson Laboratory and bred in house as heterozygous wild type crosses to provide *Apc*^*Min/+*^ mice and wild-type littermates [34]. All animals were and kept under specific pathogen-free conditions in filter-top cages. Genotyping was performed at 4 weeks by PCR [35]. Forty *Apc*^*Min/+*^ mice aged 6 weeks were randomized into 5 groups (*n* = 8 per group). The mice were provided with YYFZBJS or Aspirin for 20 weeks as previously described [36]. Briefly, the intragastric administration of YYFZBJS-L/M/H were taken at the doses of 3.825 g/kg, 7.65 g/kg and 15.3 g/kg according to HED (human equivalent dose) [32]. In the clinical practice of Chinese herbal medicine, YYFZBJS is usually prescribed at a daily dose of 51 mg of herbal materials. When this human dose was converted into an animal dose (a person of 60 kg, and a conversion factor of 9 between human and mouse), it was equivalent to the middle dose (7.65 g/kg) used in this study. Control group was oral gavaged with the same volume of sterile isotonic saline and fed with normal drinking water. The 20 week-oral gavage-protocol used in *Apc*^*Min/+*^ mice is presented in Fig. 1a Signs of illness were monitored daily and body weight was recorded weekly.

### Histology and immunohistochemistry

Mouse blood was collected from retinal venous plexus, centrifuged to harvest serum, which were stored at − 80 °C. Mice were sacrificed by cervical dislocation. The whole intestine was removed immediately after sacrifice and opened longitudinally after washed with ice-cold PBS as previously described [37]. The number, location, and size of visible tumors throughout the intestine were measured to calculate the incidence of adenoma. Tumor numbers were counted and grouped based on sizes: < 2 mm, 2–4 mm and > 4 mm. Tissue sections were fixed in 10% formalin followed by paraffin embedding. Then they were stained with hematoxylin and eosin for pathological evaluation by a pathologist blinded to the experimental groups. Histological analysis for polyp, adenoma, and adenocarcinoma was performed by a board-certified pathologist (PV) as previously described [38]. The histology scoring criteria is as follows: 0 = normal, 1 = moderate, 2 = marked and 3 = severe.

For the murine samples, immunohistochemistry was performed to detect total Ki67 (anti-mouse Ki67, Abcam), PCNA (anti-mouse PCNA, Abcam) and BrdU (anti-BrdU kit, Invitrogen); all stains used horseradish peroxidase-conjugated antibody, with chromogenic detection with the substrate 3–3′-diaminobenzidine, and finally counterstained with hematoxylin.

### Microbial analysis of mouse stool

Feces of all mice in the NS and YYFZBJS group were collected for gut microbiota analyses. Briefly, (i) genomic DNA was extracted using a PowerSoil DNA Isolation Kit (MO BIO Laboratories, Carlsbad, CA); (ii) the 16S rDNA V4 region was amplified using the 515F and 806R primers; (iii) PCR product quantification, qualification, and purification were performed; (iv) library preparation and sequencing were performed on the MiSeq platform (Illumina, Inc., San Diego, CA). The 16S rRNA sequencing data were quality filtered using FLASH (Fast Length Adjustment of Short reads, Version 1.2.11). Operational taxonomic units (OTUs) were picked at a 97% sequence similarity cut-off, and the purified amplicons were sequenced on an Illumina MiSeq platform at Majorbio Bio-pharm Technology Co. Ltd. according to the standard protocols.

### Antibiotic treatments

Mice were treated for four weeks with an antibiotic solution (Abx) containing Ampicillin (1 mg/ml), Neomycin (1 mg/ml), Metronidazole (1 mg/ml), and Vancomycin (0.5 mg/ml) added to the sterile drinking water of mice adlibitum as previously described [39]. Solutions and bottles were changed 2 times a week. After four weeks, Abx treatment was stopped and the mice were recolonized by FMT.

### Fecal microbiota transplantation (FMT)

After receiving antibiotic cocktails for 4 weeks, a volume of 200 μL suspension was gavaged to each mouse for four weeks [39]. The *Apc*^*Min/+*^ mice were divided into two groups with 8 mice each: One group was gavaged fecal samples from healthy controls (Control-FMT), while the other group was gavaged fecal samples from people who eating YYFZBJS (YYFZBJS-FMT). Each group of mice used a separate set of intragastric apparatus.

### Electron microscopic

The intestinal tissue of *Apc*^*Min/+*^ mice treatment with FMT were excised and fixed in 0.1 M phosphate buffer containing 2.5% glutaraldehyde and 2.0% paraformaldehyde (pH 7.4). Then the tissue were fixed, dehydrated, polymerized and then examined using the transmission electron microscope as previously described [40].

### Spleen to body weight ratio

Before killing, mice body weight was measured, and then mice spleens were collected, and spleen weight was measured as previously described [41]. The ratios of the spleen to body weight were calculated as spleen weight/body weight.

### Cytokine antibody arrays

Serum samples were screened in duplicates using a Mouse Cytokine Array QAM-INF-1 (RayBiotech) containing slides coated with 40 different cytokines according to the manufacturer’s guidelines with some modifications as previously described [42]. Briefly, the arrays were blocked, incubated with 100 mL of condition medium overnight, followed by biotin-conjugated antibodies (1/250) incubation for 2 h and with HRP-linked secondary antibody (1/1000) for 1 h. The membranes were incubated with a peroxidase substrate, and the results were documented using XAR films. Quantitative array analysis was performed using Array Vision Evaluation 8.0 (GE Healthcare Life Science).

### Quantitative real-time PCR and bioinformatics analyses of RNA-Seq

Tumor tissues were homogenized with 1 mL TRI reagent to extract total RNA. cDNA was synthesized by reverse transcription of total RNA (Epicentre). Quantitative real-time PCR (qRT-PCR) was carried out as previously described [43]. The Oligonucleotide primers for target genes (T-bet, Gata3, ROR-γt, Foxp3, c-Myc, Axin2, EphB3, β-catenin, TCF, LEF1, CyclinD1, Lgr5 and GAPDH) were shown in Supplementary Table S1. RNA-Seq FASTQ files were processed using the RNA-Seq module implemented in the CLC Genomics Workbench v8.0 software (Qiagen Bioinformatics) with default settings.

### Lymphocyte preparation

Spleen, mesenteric lymph nodes (MLN) and small intestine and colon, were collected from *Apc*^*Min/+*^ and WT mice. The monoplast suspension was collected by passing splenocytes through 70 μm cell strainers (BD Biosciences, Bedford, MA, USA). Red cell lysis was performed on splenic cells with 0.07 M NH_4_Cl, pH 7.3, 37 °C for 5 min. Lamina propria lymphocytes (LPL) from the small and large intestine, and from adenomas were isolated essentially as described before [44] but with the use of collagenase VIII (Sigma-Aldrich) for colon digestion.

### Analysis of cytokine expression in serum

Mouse serum samples were analyzed for mouse cytokines by ELISA according to the manufacturer’s instructions (eBioscience) and as previously described [45].

### Flow Cytometry

Phenotype analysis of Tregs was performed with a BD FACS AriaII flow cytometer (BD, USA) as previously described [46]. Briefly, the cells were labeled with CD4-FITC, CD25-APC, and Foxp3-PE (eBioscience, San Diego, CA) following the manufacturer’s protocol. To analyze the prevalence of Tregs, CD4 + Foxp3 + T cells were evaluated after gating on CD4 + T cells and were expressed as a percentage of the total CD4+ T cells.

### Preparation of Chinese YYFZBJS herb formula

The formula for creating one dose of YYFZBJS is presented in Table 1. Chinese medicines were purchased from Shanghai Hua Yu Chinese Herbs Co., Ltd. (Shanghai, China). The Chinese medicines included Yi-yi-ren (lot# 180103), Fu-Zi (lot# 180709), Bai-jiang-cao (lot# 180522). All herbs were authenticated by Associate Researcher Tao Yang according to the Pharmacopoeia of the People’s Republic of China (2015). The vouchers of all components were deposited at the herbarium located in the College of Pharmacy, Shanghai University of TCM (Shanghai, China).

**Table 1:** Formula of YYFZBJS (one dose)

Chinese medicine	Plant origin	Medicinal parts	Origin (Province)	Amount in Preparation (g)
Yi-yi-ren	Coix lacrymajobi L.var.mayuen (Roman.)Stapf	kernel	Fujian Province	211,803
Fu-Zi	Aconitum carmichaeli Debx.	root	Sichuan Province	221,304
Bai-jiang-cao	*Thlaspi arvense* Linn	aerial parts	Henan Province	201,508

All herbs were added the appropriate amount of water and then extracted twice, filtrated and dried into dry-extract according to as a validated method [32]. For quality control, the fingerprint spectrum for YYFZBJS was performed by UHPLC-Q Exactive system (Thermo, San Jose, CA, USA) equipped with a quaternary gradient pump, an autosampler, and high-resolution mass spectrometry detector. The components were eluted with a gradient system consisting of acetonitrile (A) and aqueous 0.1% formic acid (B) in gradient (time, min/B%: 0/95, 12/5,14/5,14.1/95,16/5); flow rate: 0.3 ml/min). The spectral data were recorded in the m/z range of 80–1200. Mass spectra were acquired in both negative and positive modes with ion spray voltage 3.5 kV, capillary temperature at 320 °C, auxiliary gas heater temperature at 300 °C, sheath gas (nitrogen) flow at 35 AU, auxiliary gas (nitrogen) flow at 10 AU, Scan mode: Full MS (Resolution 70,000) and dd-MS2 (Resolution 17,500, NCE35, Stepped NCE50%). The chromatographic column was ACQUITY UPLC HSS T3 (2.1 mm × 100 mm, 1.8 μm). The mobile phase flow rate was 0.3 ml/min and the column temperature was maintained at 40 °C. Otherwise, the contents of liquiritigenin, luteolin, mesalamine, aconitine, and hypaconitine were detected by UPLC-MS method and were 1.71 mg/g, 311.22 mg/g, 5.32 mg/g, 1.91 mg/g, and 74.71 mg/g in the extracts respectively.

### Network construction

The potential targets for the components of YYFZBJS were retrieved from Therapeutic Targets Database including TCMSP (http://ibts.hkbu.edu.hk/LSP/tcmsp.php), TCM database @Taiwan (http://tcm.cmu.edu.tw), and TCM Integrated Database (TCMID) (http://www.megabionet.org/tcmid). In these networks, YYFZBJS and its targets are represented as nodes, while the edges indicate interaction or relatedness (Supplementary Table S3).

### Isolation of spleen Tregs and its effect on cancer cell ability

Spleen cells from *Apc*^*Min/+*^ mice were separated over columns for negative and positive selection for CD4+ CD25+ Foxp3 MACS columns and separator (Miltenyi Biotech, CA) as previously described [47]. CD4+ CD25+ Foxp3 T cells were co-cultured with *Bacteroides fragilis* (cell: bacterial = 1:10) in RPMI-1640 medium in presence or absence of YYFZBJS (different concentrations) for 4 h and CD4+ CD25+ Foxp3 T cells were collected after centrifuging. For the analysis of Treg cell effect on cancer cell ability, the MC-38 cells were inoculated in 24 well plates with 1000 cells (per well) and co-cultured with the Treg for 12, 24, 36 and 48 h. The proliferation of MC-38 cells was measured by trypan blue as previously reported [48].

### Bacterial attachment assay

*Bacteroides fragilis* (43858) were purchased from ATCC and cultured in lysogeny broth at 37 °C. The bacterial attachment assay was performed as described previously [49]; Treg cells were co-cultured with bacteria for 4 h (MOI = 10) under anaerobic conditions. After co-culture, medium was removed and cells were washed with PBS three times. Then cells were lysed, and added Wilkins-Chalgren anaerobe broth to homogenize. The attached *Enterotoxigenic Bacteroides fragilis* (ETBF) colonies were recovered on Wilkins-Chalgren anaerobe agar plate under anaerobic conditions; the number of colonies was counted.

### Western blot analysis

Whole cell lysates for Western blot analysis of β-catenin (nuclear, cytoplasm), PCNA and β-actin expression were prepared as previously reported [42]. Briefly, total lysates from treated cells were prepared with RIPA buffer (50 mM Tris, pH 7.2; 150 mM NaCl; 0.5% sodium deoxycholate; 0.1% sodium dodecyl sulfate; 1% Nonidet P-40; 10 mM NaF; 1 mM Na3VO4; protease inhibitor cocktail. Lysates were sonicated for 10 s and centrifuged at 14,000 rpm for 10 min at 4 °C. Protein concentration was determined by bicinchoninic acid assay with BSA as a standard (Pierce, Rockford, IL, USA). Equivalent amounts of protein (50 μg/lane) were separated on 7.5–12% SDS-polyacrylamide gel and transferred to polyvinylidene difluoride membranes (Millipore, Bedford, MA, USA). Membranes were incubated with PBS containing 0.05% Tween 20 and 5% nonfat dry milk to block nonspecific binding and were incubated with primary antibodies, then with appropriate secondary antibodies conjugated to horseradish peroxidase. Immunoreactive bands were visualized by using Renaissance chemiluminescence reagent (Perkin-Elmer Life Science, Boston, MA, USA). Densitometric analysis was performed using the Scion Imaging application (Scion Corporation), with β-actin as the internal reference.

## Results

### YYFZBJS suppresses intestinal tumorigenesis and expression of Ki67, PCNA, and reactivity to BrdU in the Apc^Min/+^ mouse model

Previously, we showed that traditional Chinese herbs were sufficient to inhibit colorectal carcinoma multidrug resistance (MDR) in nude mouse [28, 29]. In the present study, we first sought to determine whether traditional Chinese herbs were beneficial for innate immunity and intestinal tumorigenesis in *Apc*^*Min/+*^ mice. The structures of the determined experiment from the herbs are shown in Fig. 1a. Consistent with the clinical results, no difference was noted in animal weight and hepatorenal toxicity among treatment groups during the experiment (Supplementary Fig. 1&2), meaning the herbs were safe for the general health of the animals. Following a 20 weeks intragastric administration of aspirin, *Apc*^*Min/+*^ mice contained much less intestinal adenomas compared with that in the no-aspirin group (Fig. 1b&c and Supplementary Fig. 3). Similarly, we discovered that *Apc*^*Min/+*^ mice treated with YYFZBJS carried fewer adenomas both in the small and large intestine (Fig. 1c&d&e and Supplementary Fig. 4), compared with the normal saline Controls(control). Notably, the numbers of polyps in all three YYFZBJS groups were all much fewer than that of the non-treated control group (Fig. 1e).

Control tumors in untreated *Apc*^*Min/+*^ mice were histologically identified as polyps with severe atypia or early carcinoma with submucosal infiltration. Notably, early carcinomas in the colon were completely eradicated by YYFZBJS treatment (Fig. 1f). The poly adenomas in small intestine, featuring moderate mucosal epithelium architectural changes with some budding and branching, was much fewer in number in YYFZBJS treated *Apc*^*Min/+*^ mice than the untreated ones (Fig. 1f). However, high-grade dysplasia adenocarcinoma (including early carcinoma), was only found in 0% (0/8), 25% (2/8), 37.5% (3/8) of mice in high-, middle-, and low-YYFZBJS dose-treated *Apc*^*Min/+*^ mice compared to 100% (8/8) of untreated mice (Supplementary Table S2). These data indicate that YYFZBJS slows down the intestinal adenoma-to-adenocarcinoma progression.

Since Ki67, PCNA and BrdU are cell proliferation markers, we then examined the localization and expression levels of Ki67 and PCNA by immunohistochemistry in the tumors of *Apc*^*Min/+*^ mice with or without YYFZBJS treatment (Fig. 1g&h). Comparing with the untreated mice, nuclear expression levels of Ki67 and PCNA, and BrdU reactivity in intestinal polyp epithelia were reduced after YYFZBJS treatment (Fig. 1g, h).

### YYFZBJS modulates the gut microbiome composition

**Fig. 2:**
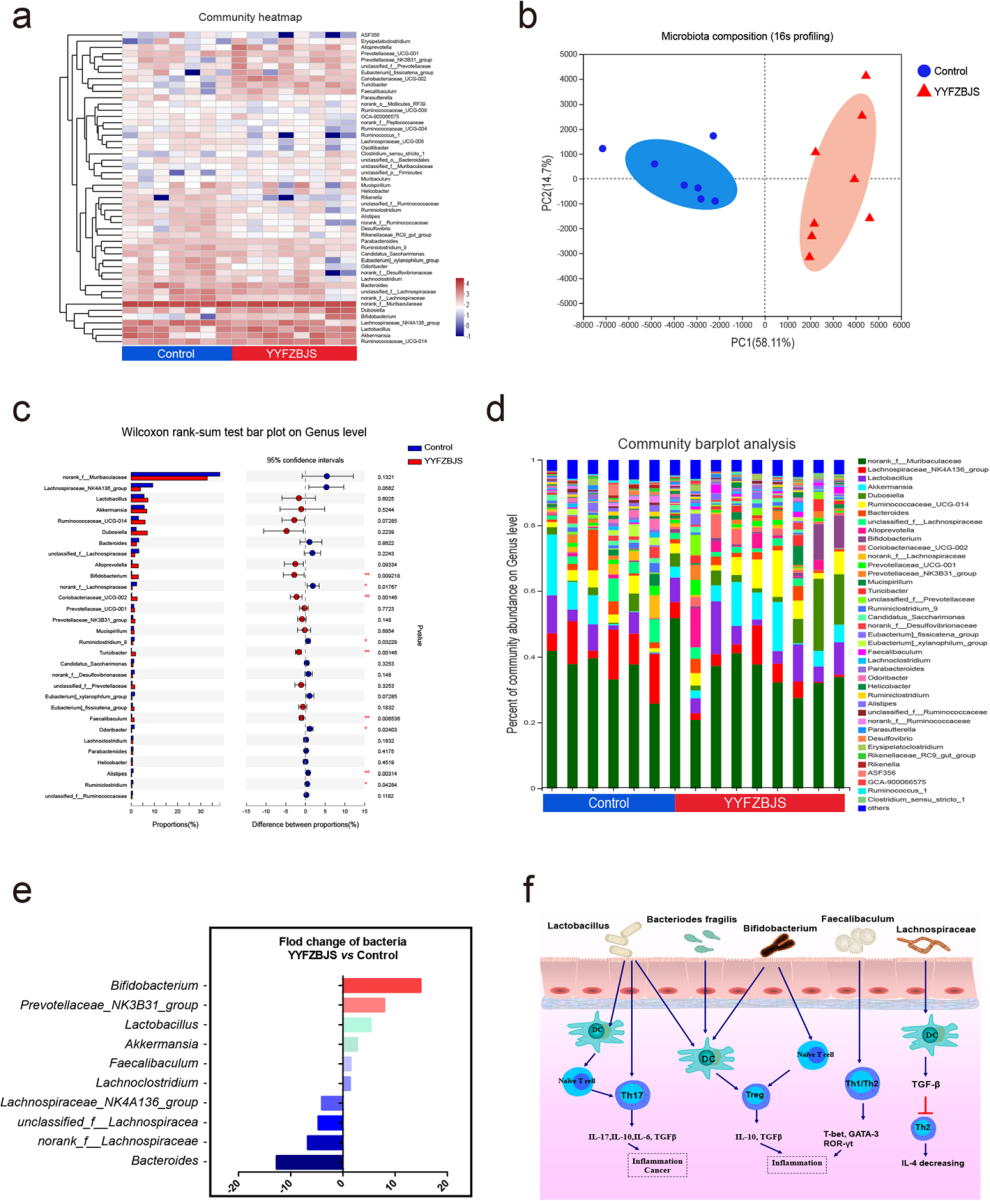
YYFZBJS modulates the gut microbiome composition. **a** Heat map of Genus with relative abundances that are significantly different from their relative abundances at the time of YYFZBJS administration. The differentially enriched bacterial Genus in C57BL/6 J mice receiving N. S and YYFZBJS. The relative abundance between control and treatment mice for the genus was calculated for each time. Blue boxes indicate negative associations (*n* = 7) and red boxes indicate positive associations (*n* = 8). **b** Principle component analysis (PCA) analysis at the genus-level, which was used to study the differences in the composition of bacterial communities in the fecal samples between mice treated with YYFZBJS and the Control group. Samples along PC1 (x-axis) explained 58.11% and PC2 (y-axis) explained 14.7% of variability, respectively. **c** Bar plot of compositional differences at the genus level in the gut microbiome of mice in the combination YYFZBJS group vs. the control group by the Wilcoxon rank-sum test. Data are expressed as mean ± SD. * 0.01 < *P* ≤ 0.05, ** 0.001 < *P* ≤ 0.01, *** *P* ≤ 0.001, Two-sided Hypotheses. **d** A stacked bar plot of genus-level phylogenetic classification of 16S rRNA frequencies in stool pellets collected from naive animals (N.S; n = 7), Chinese herb decoction-treated animals (YYFZBJS; n = 8). **e** Relative fold change of the 10 most abundant bacterial families abundances, which was significantly different between mice treated with YYFZBJS and the Control group. **f** The gut microbiome has a profound effect on the host immune system, including DCs, naive T cells, Tregs and Th17 cells. The relationship between the five types of bacteria and immune cells is summarized

We next sought to characterize the effects of YYFZBJS treatment on intestinal bacterial communities through analysis of bacterial 16S rRNA compositions. In the *Apc*^*Min/+*^ mice model, YYFZBJS and NS group developed different gut microbiota: we observed a significantly lower bacterial richness in the YYFZBJS group (Fig. 2a). Nonmetric multidimensional scaling analysis demonstrated the clear separation of bacterial OTU composition (Fig. 2b). Alterations at the genus level were also assessed (Fig. 2c). While a significant elevation in abundance of several probiotic genera (*Bifidobacterium* and *Prevotellaceae*) was determined in response to YYFZBJS treatment, some genera (*Bacteroides, Lachnospiraceae*, *unclassified lachnospiraceae among* others) were nearly eliminated. YYFZBJS administration resulted in reduced frequencies of bacteria belonging to the *Firmicutes*, including *Lactobacillus* and *Dubosiella* (Fig. 2d). Consistent with the results of species richness, YYFZBJS administration selectively blunted the relative expression of the *Bacteroides*, *Lachnospiraceae* and so on (show significant changes in top 10) (Fig. 2e). Furthermore, based on the published studies and our screening results, the mechanisms of concentrated bacteria in regulating CD4+ T cell-derived effectors were demonstrated in regulating host immunity (Fig. 2f).

### Gut microbiota from YYFZBJS users delay the progression of intestinal tumorigenesis

**Fig. 3:**
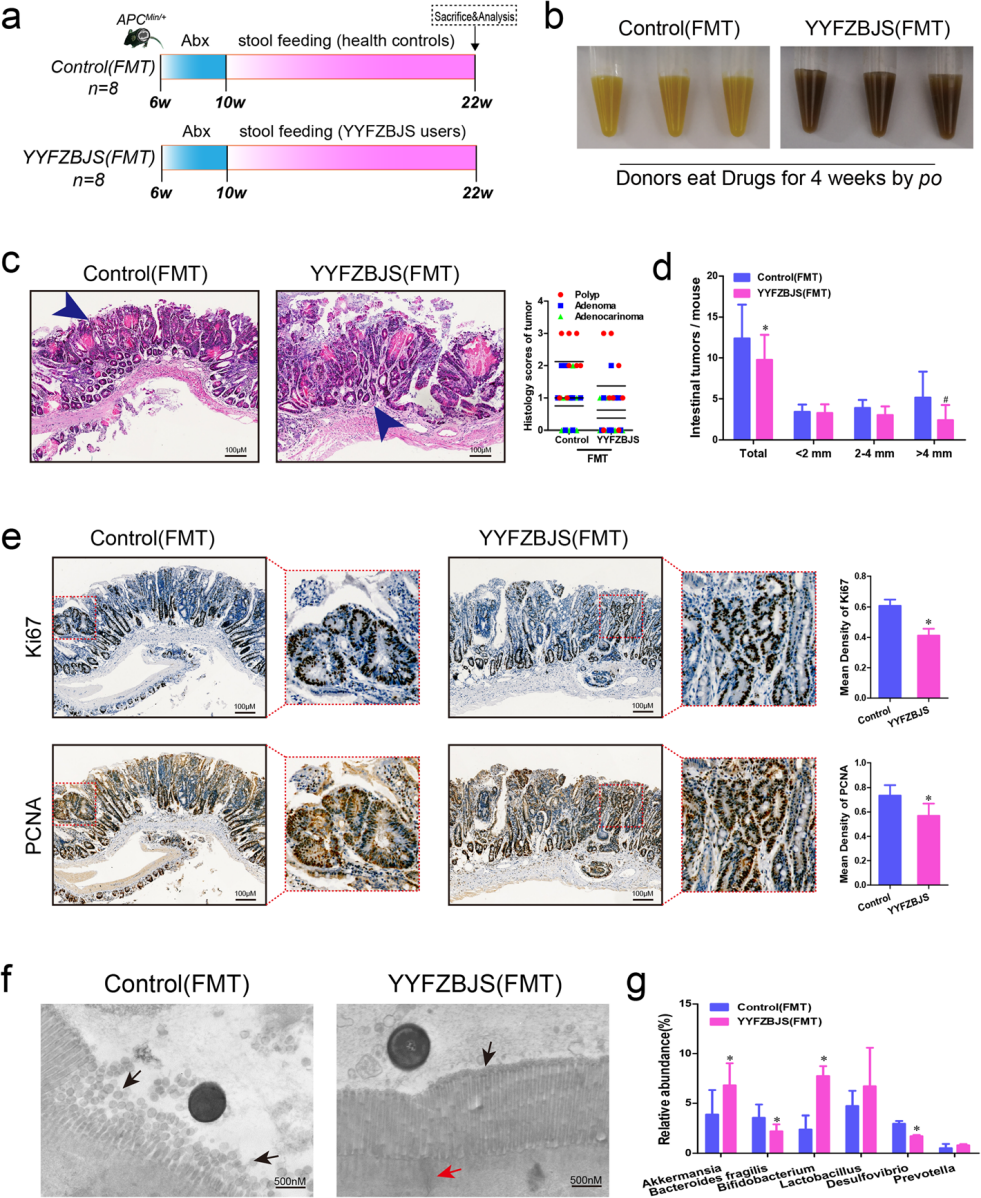
Gut microbiota from YYFZBJS volunteers delay the progression of intestinal tumorigenesis. **a** Design of stool gavage experiment to *Apc*^*Min/+*^ mice. Mice were treatmented with Abx from week 6, and sacrificed at week 22 (n = 8). **b** Display of the fecal extracts of the *Apc*^*Min/+*^ mice with FMT treatment for 12 weeks. **c** Left: typical adenomatous polyp seen in infected *Apc*^*Min/+*^ mice showing high-grade dysplasia and carcinoma in situ. Right: minute polyp with remnant dysplastic glands close to the surface epithelium. Blue arrows indicated adenocarcinoma cell. Magnification bars, 100 μM. Histological analysis of intestinal tumors applyed in the two FMT group mice (n = 8 for each group). **d** The tumor size distribution in the intestine was listed and compared with control-FMT (n = 8 for each group). Data shown represent means ± SD. *P < 0.05 vs. control-FMT. **e** Immunohistochemical staining with an antibody against PCNA and Ki67 in control-FMT group and YYFZBJS-FMT treatment group. Magnification bars, 100 μM. Data are given as means ± SD of 8 animals per experimental group, with Welch’s correction, one-tailed t-test. *P < 0.05 vs. control-FMT. **f** Electron microscopy in the lumen infiltration of control-FMT group mice and YYFZBJS-FMT mice at age of week 22. Both microvilli and goblet cells can also be seen. The black arrow refers to the intestinal microvilli; The red arrow indicates a tight connection. Magnification bars, 500 nM. **g** Fecal bacterial DNA was prepared from Control-FMT group and YYFZBJS-FMT treatment group. Relative genus abundance was shown as percentage of each OTU in the total OTUs (*n* = 5/group). Data shown represent means ± SD. *P < 0.05

The impact of FMT with or without YYFZBJS on intestinal adenoma was evaluated after treatment with 12 weeks (Fig. 3a&b). No significant gross bloody stool was observed in the two groups (data not shown). All *Apc*^*Min/+*^ mice were well tolerated and survive after FMT. In the YYFZBJS-FMT group, a few scattered small polyps were observed, while more adenomas were observed in the Control-FMT group, especially the cribriform morphology appeared in the tumors (Fig. 3c&e). The total number of intestinal tumors in mice receiving fecal samples from YYFZBJS volunteers was decreased compared with the health controls (Fig. 3d). Comparing with the Control-FMT group, the rate of Ki-67 and PCNA positive cells in YYFZBJS-FMT group was significantly decreased (Fig. 3e). Our EM imaging data indicate that microvilli with lodging, fracture and fall off were observed in the intestinal mucosal ultrastructure of Control-FMT group (Fig. 3f, left panel), comparing with most epithelium microvilli arranged closely neat and orderly, which can be seen in the lumen infiltration of YYFZBJS-FMT group (Fig. 3f, right panel). Consistent with the changes in flora of *Apc*^*Min/+*^ mice which suffer from YYFZBJS administration, some intestinal bacterial have been regulated significantly after YYFZBJS-FMT treatment, such as Bifidobacterium, Akkermansia, Lactobacillus, Desulfovibrio, Bacteroides and Prevotella (Fig. 3g). Therefore, the results suggested that gut microbiota from YYFZBJS users inhibited the progression of intestinal adenoma in *Apc*^*Min/+*^ mice.

### Effect of YYFZBJS on immunity of Apc^Min/+^ mice

**Fig. 4:**
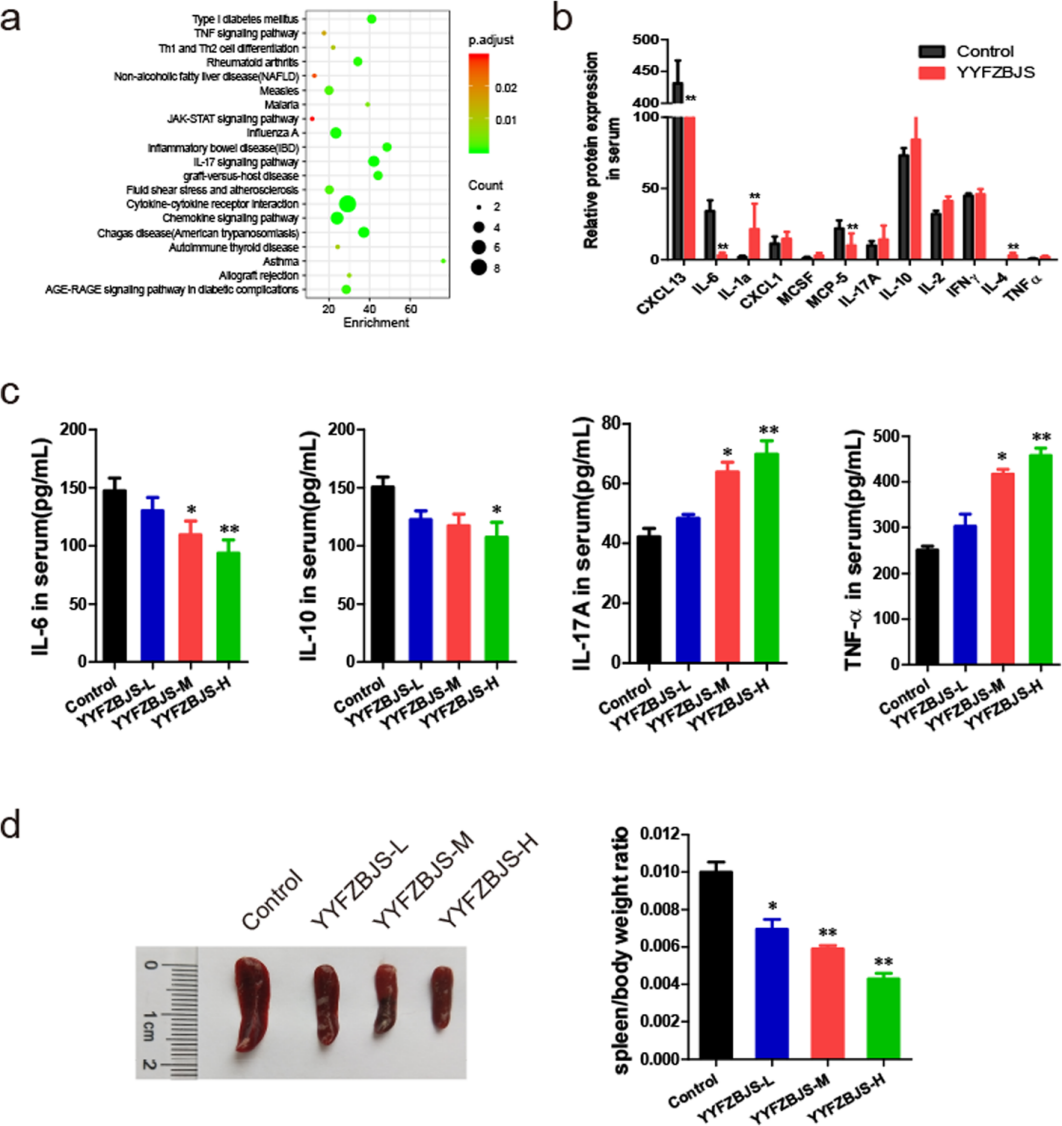
Effect of YYFZBJS on the immunity of *Apc*^*Min/+*^ mice. **a** The difference between the two groups in inflammatory cytokines as assessed by cytokine antibody array. **b** Top common genes that are regulated significantly after treatment with YYFZBJS. **c** IL-6, IL-10, IL-17A and TNF-α levels in PBMC of *Apc*^*Min/+*^ were evaluated using ELISA. **d** Representative pictures of spleens from *Apc*^*Min/+*^ mice that were orally treated with YYFZBJS for 20 weeks. Spleen weight of *Apc*^*Min/+*^ mice was assessed on the right. The data are presented as the mean ± SD from at least three experiments. *P < 0.05, **P < 0.01 vs. control

To determine whether YYFZBJS changed levels of inflammatory cytokines, we used a cytokine antibody array to illustrate that, compared to normal control mice, *Apc*^*Min/+*^ mice secreted higher levels of inflammatory cytokines/chemokines including IL-17, Eotaxin-2, Leptin and PF4 (Supplementary Fig. 5). It was previously demonstrated that YYFZBJS negatively regulated inflammatory cytokines IL-17, and IL-10 in myeloid precursor differentiation [50]. Consistent with that, YYFZBJS treated mice expressed lower level of IL-6, CCXL13 and IL-10 than the untreated group, and suggesting Treg cells were probably changed by YYFZBJS treatment (Fig. 4a&b).

In consistency with the qRT-PCR data, further results of ELISA showed that the expression of IL-6 and IL-10 in PBMC of *Apc*^*Min/+*^ mice was significantly decreased by YYFZBJS treatment, whilst IL-17A and TNF-α were upregulated (Fig. 4c). Splenomegaly, one of the prognostic characteristics that correlate with intestinal tumor progression in *Apc*^*Min/+*^ mice, was also decreased by YYFZBJS: a ~ 57% reduction in the spleen weight index compared to the untreated controls (Fig. 4d). These results suggested that YYFZBJS blocked tumor progression in CRC murine model possibly via inhibiting the accumulation of Treg cells in immune organs, and in tumor microenvironment.

### YYFZBJS modulates the function of regulatory T cells in MLN, spleen, LPL, and PBMC of Apc^Min/+^ mice

**Fig. 5:**
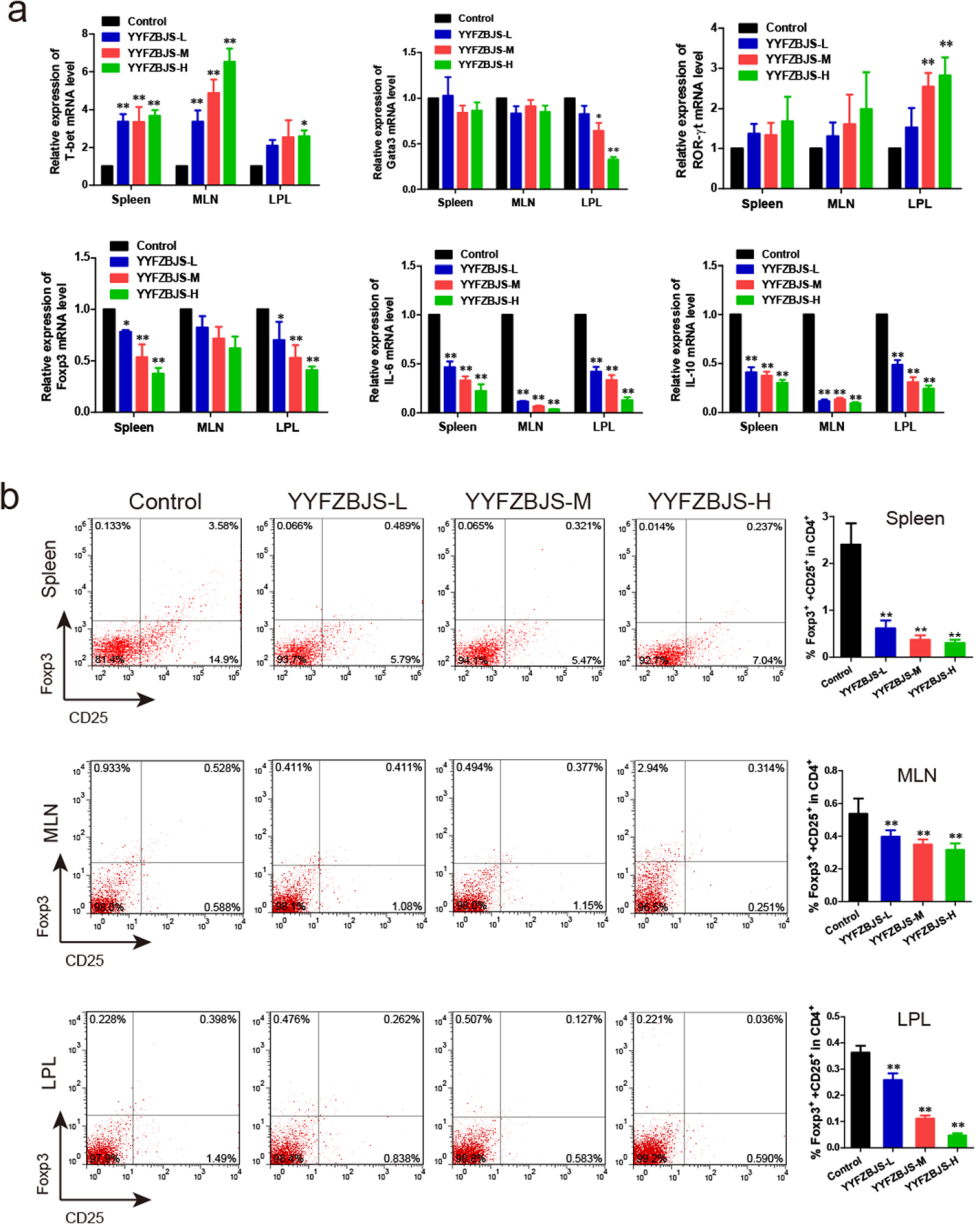
Effect of YYFZBJS on mRNA expression of TH cell in the spleen, MLN and LPL of *Apc*^*Min/+*^ mice. **a** Foxp3, Gata3, ROR-γt, and T-bet expression in polyp were evaluated using RT-PCR. **b** Representative flow cytometry plots of intestinal lamina propria cells (LPCs) showing the viable lymphocyte gate on CD4 + T cells. Representative plots showed the frequency of CD25+ Foxp3+ T cells after drug intervention for 20 weeks in the intestinal lamina propria of *Apc*^*Min/+*^, as determined by flow cytometric analysis. The data are presented as the mean ± SD from at least three experiments. *P < 0.05, **P < 0.01 vs. control

As observed previously, a significantly alternative frequency of Helper T lymphocytes was observed in the splenomegaly animal mode, which has been linked to unfavorable clinicopathological features and poor prognosis [12]. We then determined the effect of YYFZBJS on the mRNA expression of T-bet, Gata3, ROR-γt, and Foxp3, which are considered as the master regulator of Helper T lymphocytes development and function in the immune system of mice. Notably, the increased Foxp3, IL-6 and IL-10 mRNA levels of LPL, spleen, and MLN lymphocytes in *Apc*^*Min/+*^ mice was offset by YYFZBJS treatment in a dose-dependent manner (Fig. 5a). These results lend support to the notion that Tregs and Th17 cells may contribute to tumor progression and can be potential therapeutic targets.

We then investigated the abundance of CD4+, CD25+, and Foxp3+ T cells within total T cell population in spleen, MLN, LPL in mice treated with YYFZBJS. YYFZBJS decreased Foxp3+ population in CD25+/CD4+ T cells in a dose-dependent manner (Fig. 5b). However, there appeared to be no significant difference in the abundances of CD4+ and IL-17+ T cells in spleen lymphocytes between untreated and YYFZBJS groups (Supplementary Fig. 6), which is consistent with what in the lymphocytes of MLN and LPL (data not shown).

### YYFZBJS inhibited tumor cell proliferation through regulating ETBF primed Treg in vitro

**Fig. 6:**
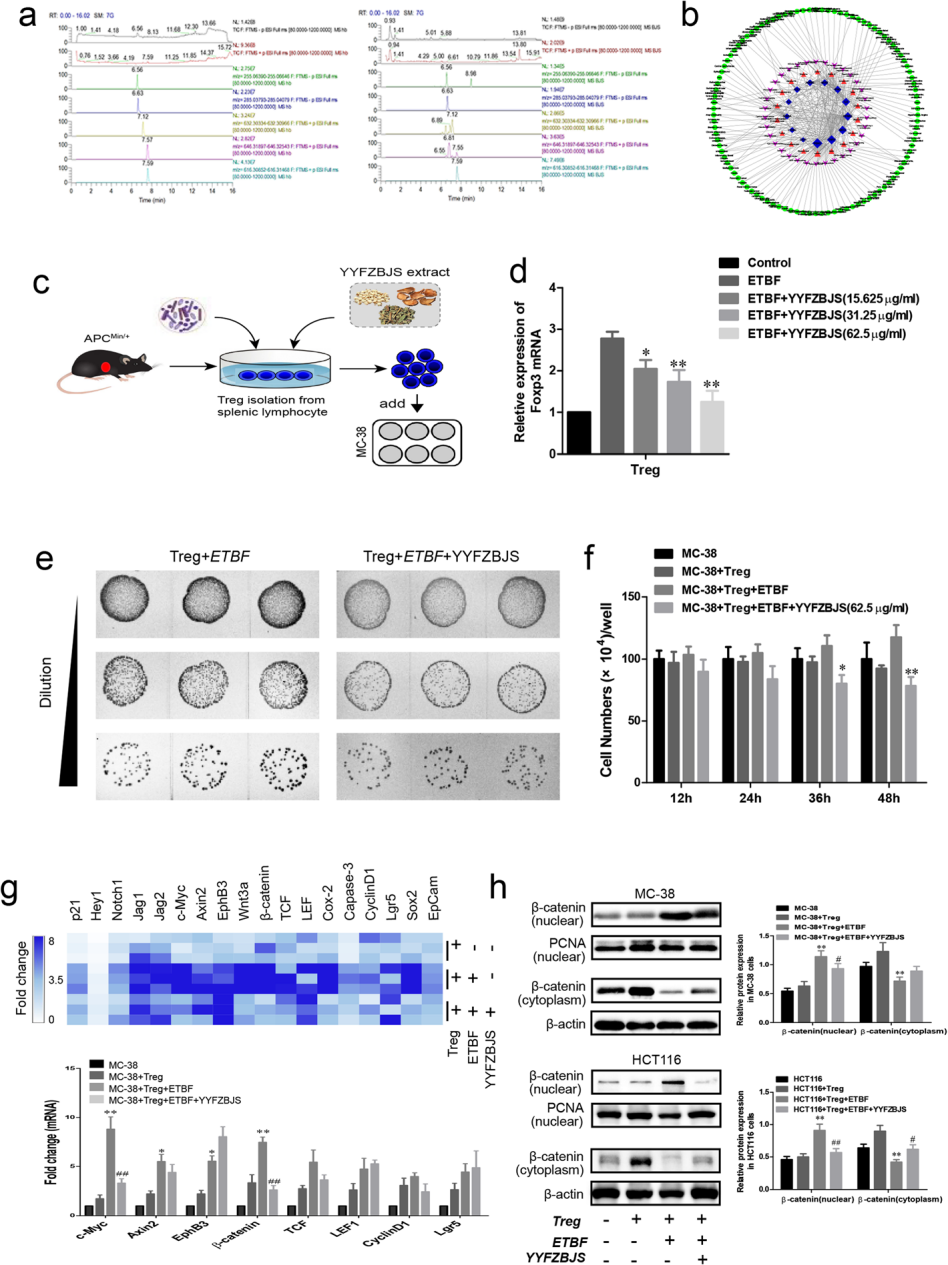
YYFZBJS inhibited tumor cell proliferation through regulating Treg in vitro*.***a** Left: YYFZBJS extracts samples, 25.66 mg/ml; Right: the mix standard solution; Stationary phase: ACQUITY UPLC HSS T3 (2.1 mm × 100 mm, 1.8 μm); mobile phase: acetonitrile (**a**) and aqueous 0.1% formic acid (**b**) in gradient (time, min/B%: 0/95, 12/5,14/5,14.1/95,16/5); flow rate: 0.3 ml/min); column temperature:45 °C]. R:6.56 min liquiritigenin ([M-H]-,255.09518 m/z); 6.63 min, luteolin ([M-H]-,285.03936 m/z); 7.12 min mesaconine ([M + H]-,632.3065 m/z); 7.55 min, aconitine ([M + H]-,632.3065 m/z); hypaconitine 7.59 min ([M + H]-,632.3065 m/z). **b** Interaction network diagram between the active ingredients of YYFZBJS and their targets using prediction software of Cytoscape 3.6.1. **c** Experimental design indicating CD4 + CD25 + Foxp3+ T cells (Treg) were isolated from spleens of *Apc*^*Min/+*^ mice treated with or without ETBF and/or YYFSBJS in 62.5 μg/ml for 4 h. The ratio of cell to bacterial was 1:10. Then the primed Treg was collected and were assigned to MC-38 cells as 10:1 ratio. **d** Foxp3 mRNA expression was analyzed by real-time polymerase chain reaction analysis in Treg cells. The data are presented as the mean ± SD from at least three experiments. *P < 0.05, **P < 0.01 vs. ETBF group. **e** The decrease in ETBF count was observed in Treg incubated with ETBF and YYFZBJS (62.5 μg/ml) group. The representative gut bacteria also had higher colony-forming unit per milliliter as observed from agar plates. **f** MC-38 cells proliferation was assayed at 0, 12, 24, 36, and 48 h after co-culture with the primed Treg. The data are presented as the mean ± SD from at least three experiments. *P < 0.05, **P < 0.01 vs. MC-38 + Treg+ETBF. **g** The heatmap displays relative fold changes in expression levels normalized to the mean expression in the Control, Treg incubated with MC-38 cells (Treg: MC-38 = 10:1), ETBF primed Treg incubated with MC-38 cells, and YYFZBJS (62.5 μg/ml) combined with ETBF primed Treg incubated with MC-38 cells for each indicated mRNA of MC-38 cell. The color brightness of each unit is associated with differences. Blue color represents high expression and white color represents low expression. Not all the mRNAs in the figure were labeled. *P < 0.05, **P < 0.01 vs. MC-38 + Treg; ^##^*P* < 0.01 vs. MC-38 + Treg+ETBF. **h** Western blot and quantitative assay of β-catenin (nuclear, cytoplasm) in MC-38 cells. β-actin as loading control The data are presented as the mean ± SD from at least three experiments. **P < 0.01 vs. MC-38 + Treg; ^#^*P* < 0.05, ^##^*P* < 0.01 vs. MC-38 + Treg+ETBF

Based on the results in the *Apc*^*Min/+*^ mouse model, we attempted to explore the anti-tumor mechanism of YYFZBJS in vitro. Firstly, YYFZBJS was analyzed by UPLC-MS, a simple and accurate HPLC method for the simultaneous separation and identification of five components for functional evaluation (Fig. 6a). As shown in the previous study [51], a comprehensive approach was employed to clarify the synergistic effects and mechanisms of multi-component, multi-target agents in YYFZBJS, and this approach included combined prediction of active compounds and identification of multiple drug targets by network analysis. To explore the potential immune mechanisms in YYFZBJS, we further identified 13 inflammation, immune-related targets, 34 pathway and 103 varieties of disease for 18 effective constituents from YYFZBJS. Based on KEGG pathway enrichment, we found that the T-cell receptor and Toll-like receptor signaling pathways were significantly affected (Fig. 6b). Therefore, we postulated that YYFZBJS exerted therapeutic effects on multiple targets and pathways, including the ones that regulate immunity, through its active components.

Before exploring the activity of YYFZBJS extract on CRC cell proliferation, we measured its cytotoxicity on MC-38 and HCT116 cells, to find that YYFZBJS had minimal effect on the cell viability of these cells (Supplementary Fig. 7). The growth of the cell is in an unaffected state when the concentration of YYFZBJS extract below 62.5 μg/ml. To determine whether the presence of gut commensal bacteria affects regulatory T cells in vitro, *Enterotoxigenic Bacteroides fragilis* (ETBF) were co-incubated with CD25+/CD4+ T cells which isolated from the spleens of *Apc*^*Min/+*^ mice (Fig. 6c). qPCR Results showed the mRNA of Foxp3 were increased when ETBF were present in CD25+/CD4+ T cells, but this phenomenon was inhibited by YYFZBJS extract at 15.625 μg/ml, 31.25 μg/ml, and 62.5 μg/ml (Fig. 6d). The result of Fig. 6e displayed significantly lower colony-forming unit per milliliter compared to controls when incubated with YYFZBJS extract. Afterwards, treated Tregs were collected and added into MC-38 cells as processed in Fig. 6c. Cell viability assays indicated, interestingly, ETBF primed Treg enhanced the proliferation of MC-38 cells, in spite of lacking direct effect on cancer cells by itself (Fig. 6f). Similar as the results in Fig. 6d, compared with the ETBF primed Treg group, the proliferation of MC-38 cells were decreased after treated with YYFZBJS extract (62.5 μg/ml) and ETBF primed Treg (Fig. 6f). To explore underlying mechanisms of these ETBF -Treg phenomena on CRC cells, we determined expression levels of proliferation markers of cancer cell and stem cell in MC-38 cells, and found a significant decrease in the number of cancer stem cell positive for mRNA expression in the YYFZBJS extract (62.5 μg/ml) and ETBF primed Treg group (Fig. 6g). Based on the above mRNA results, the changes in protein expression of β-catenin (nuclear, cytoplasm and total) were analyzed by Western blotting in both MC-38 cells and HCT116 cells. ETBF primed Treg could significantly increase the nuclear accumulation of β-catenin, while YYFZBJS extract (62.5 μg/ml) combined with ETBF primed Treg could effectively reverse the distribution of β-catenin in the nucleus (Fig. 6h). Consistent with our previous research, the change of β-catenin aggregation in the nucleus is still the characteristics of Wnt signaling pathway activation [52].

## Discussion

Epidemiologic studies suggest that herbal medicines reduce colon cancer risk in humans [53]. Here, we showed that YYFZBJS, a traditional Chinese herbal medicine from Synopsis of Golden Chamber, significantly reduced tumor multiplicity and numbers in the *Apc*^*Min/+*^ CRC mouse model. Previous studies have confirmed that all three components of YYFZBJS have potent anti-cancer effect by inhibiting intestinal tumor formation [28–30]. Other recent findings have confirmed that, coix seed and patrinia, the major active constituents of YYFZBJS, regulate T lymphocytes and improve immunity [54, 55]. In our current study, we used a simple and accurate UPLC-MS technology to simultaneously separate and identify three drug components to evaluate YYFZBJS.

Emerging evidence suggests that gut microbiota, along with immune and metabolite factors, contribute to CRC carcinogenesis [4, 56]. We found YYFZBJS treatment changed bacterial taxa in the colon of *Apc*^*Min/+*^ mice. OTUs results further showed that the bacteria of *Lactobacillus*, *Dubosiella*, might play an active role in both pro- and anti-inflammatory T-cell regulatory pathways. Interestingly, several studies have highlighted the inducement of colon Treg cells is closely related to the gut microbiome [2, 6]. Reports showed that Treg cells can be upregulated by certain bacterial strains and metabolic substances from *B. fragilis* [6, 57]. As expected, our data also found that YYFZBJS FMT administration modulates microbial consortia on colorectal carcinogenesis and results in a significant reduction in overall polyp number and size. It also showed superiority in restoring gut microbiota diversity, which suggested that the anti-tumorigenesis effect of YYFZBJS was mediated mainly through the complex microbiome.

Several studies have confirmed that gut microbiota from CRC patients showed intestinal mucosal barrier damage, low grade intestinal inflammation, activation of adenomas progression [2, 7]. Previous research has shown that FMT restored both the ratio and diversity of gut microbiota, which promoted the CD4 + CD25 + Foxp3+ cells and attenuated T helper (Th)1/2/17 cells in CAC mice [6]. Similar to our bacteria analysis results, our microarray data suggest that the YYFZBJS evoke multiple inflammatory and oncogenic pathways in CRC carcinogenesis, especially on Treg/Th17 signaling because of significant impacts on IL-6, IL-10, IL-17 expression. Several studies have shown the Treg involvement in colorectal tumorigenesis, e.g. IL-6 and IL-10 both enhanced tumorigenesis in colitis-associated cancer models [58, 59], whereas blockade of IL17A inhibited tumor growth [28]. In animal experiments of colorectal carcinogenesis, mechanisms of the effects of microbiota on immune homeostasis have been studied extensively, with some studies demonstrating that *Lactobacillu*s or *Bacteroides fragilis* coordinate Treg/Th17 balance to regulate carcinogenesis [16, 22].

Accumulating data also indicated that the percentage of Treg cells is inversely related to increasing the risk for the progression of cancer [60, 61]. For CRC patients, increased numbers of Treg cells had been found in peripheral blood, tumor-draining lymph node (DLN), and tumor microenvironment. Coincidently, Tregs have also been reported by clinical observations and mechanistic studies, to play an indispensable role as a promoter of tumor growth because of its suppressive effects on the autologous effector T-cell responses. However, others found that Tregs may inhibit the intestinal tumor growth in adenomatous polyposis coli (*Apc*)-mice [62, 63]. Nonetheless, in the early stages of tumor development, it is widely understood that the balance of lymphocyte-recruiting chemokines is altered, possibly contributing to the observed shift toward higher abundance of Treg. What is more, Treg can inhibit the function of effector T helper cells and cytotoxic T cells, and also act on antigen presenting cells to reduce their capacity to activate naive T cells [64]. Recently, our group described an important involvement of the immune response in DSS-induced colitis in mice through regulating Treg cell stability and function, to promote cancer development [65].

In order to confirm our hypothesis that immune responses are responsible for the anticancer activities of YYFZBJS, local lymphocyte accumulation in adenomas was examined. YYFZBJS decreased expression levels of Foxp3, IL-6 and IL-10 in conventional T cells in adenomas. In the early stage of the disease, Treg cells and their effect molecule IL-10 serve an important, protective role against cancer by maintaining immune homeostasis [56]. Therefore, as most clinical studies have found, high intra-tumor Treg abundance correlate with improved outcome in CRC [66, 67]. Consistent with these observations, we found the expression of Treg associated cytokines such as TNF-α, IL-6, IL-17A, and IL-10 was dysregulated in *Apc*^*Min/+*^ mouse. In support of this, abundance of CD4+ T Foxp3 Tregs was significantly reduced, especially in the lymphocytes of LPL, by YYFZBJS.

To explore the anti-tumor mechanism of YYFZBJS in the internal environment, it is the first time that the herbal extracts has been co-cultured with gut microbiota and T cells in our study (Fig. 6c). Interestingly, YYFZBJS showed insignificant changes in the cell viability of CRC HCT116 and MC-38 cells. However, the anti-proliferative effect of YYFZBJS (the same dose) was significantly enhanced through ETBF primed Tregs incubated with MC-38 cells. Further experiments indicated that the altered Tregs mediated by YYFZBJS could inhibit cancer cell proliferation by alteration of nuclear β-catenin in cancer cells [52], which have been taken as the clinically crucial role in the activation of Wnt/β-catenin signaling pathway. Of note, we hypothesized that CRC carcinogenesis is due to unmitigated inflammatory response and upregulation of immune cell in intestinal tissues. Since the effect of YYFZBJS in nude mouse, which lacks T cell immunity, was not obvious (data not shown).

There remain some limitations to mention in this work. ETBF is not the only microbial regulated by YYFZBJS, the other gut microbiome also play an important role in the development of the colorectal cancer. However, our research is only a initial exploration of the mechanism of YYFZBJS in the complex microbiome, and no in-depth studies have been conducted on other bacterial groups in vitro. Also, due to the limitation of current testing methods, we were not able to continually monitor the dynamic and interactive changes of gut microbiota, which indeed require further researches.

## Conclusions

The present study reports for the first time that YYFZBJS markedly delays the progression of CRC in *Apc*^*Min/+*^ mice. The observed effects were supported by the tumor load change and gut tissue histology. Specifically, we demonstrate that growth of cancer cells can be influenced by the commensal microbiota via Treg cell induction. This was supported by the fact that YYFZBJS treated lymphocyte–conditioned medium (LCM) inhibited MC-38 tumor cell proliferation through inhibiting the phosphorylation of β-catenin.

This discovery helps us better understand the anticancer effect of YYFZBJS and its ability to remodel the gut microbiota, leading to regulation of immunity and delay of carcinogenesis. Future studies will address functional significance of loss of the Treg in the *Apc*^*Min/+*^ intestinal tumor microenvironment, to pave a way for the use of YYFZBJS in CRC immunotherapy.

## Supplementary information

**Additional file 1: Table S1.** PCR primers. **Table S2.** Histopathologic analysis of neoplastic lesions and the degree of dysplasia. **Table S4.** Clinical characteristics of the human donors for stool gavage to mice. **Figure S1.** The effect of YYFZBJS on body weights of *Apc*^*Min/+*^ mice. **Figure S2.** The effects of YYFZBJS on the liver and kidney in *Apc*^*Min/+*^mice. **Figure S3.** The effects of YYFZBJS in intestinal tumorigenesis. **Figure S4.** The effects of YYFZBJS in intestinal tumor numbers. **Figure S5**. Heatmap of inflammatory cytokines analyses between C57BL/6 J mice and *Apc*^*Min/+*^ mice. **Figure S6**. The phenotype of IL-17-producing T cells Th17 in the spleen of *Apc*^*Min/+*^ mice was examined. **Figure S7.** The effect of YYFZBJS on CRC cell proliferation.

**Additional file 2: Table S3.**

## Data Availability

All data generated or analyzed during this study are included in this published article.

## Abbreviations

CRC: Colorectal cancer
YYFZBJS: Yi-Yi-Fu-Zi-Bai-Jiang-San
FMT: Fecal microbiota transplantation
PBMC: Peripheral blood mononuclear cell
PCNA: Proliferating Cell Nuclear Antigen
IFN-γ: interferon gamma
IL-6/10: interleukin-6/10
TNF-α: tumor necrosis factor-α
*Apc*: adenomatous polyposis coli
BrdU: 5-Bromo-2-deoxyuridine
ACF: aberrant crypt foce
AOM: Azoxymethane
TCM: Traditional Chinese Medicine
MLN: mesenteric lymph nodes
LPL: Lamina propria lymphocytes
qRT-PCR: real time quantitative reverse transcription PCR
MDR: multidrug resistance
DLN: draining lymph node
LCM: lymphocyte–conditioned medium
NS: normal saline
EM: Electron microscopy
ETBF: *Enterotoxigenic Bacteroides fragilis*
