# Supplementary material for: YYFZBJS ameliorates colorectal cancer progression in ApcMin/+ mice by remodeling gut microbiota and inhibiting regulatory T-cell generation
Source: Cell Commun Signal. 2020 Jul 16;18:113. doi: 10.1186/s12964-020-00596-9 (PMC7367414; doi:10.1186/s12964-020-00596-9)
Supplement: Supplementary file 2 — Additional file 1: Table S1. PCR primers. Table S2. Histopathologic analysis of neoplastic lesions and the degree of dysplasia. Table S4. Clinical characteristics of the human donors for stool gavage to mice. Figure S1. The effect of YYFZBJS on body weights of ApcMin/+ mice. Figure S2. The effects of YYFZBJS on the liver and kidney in ApcMin/+mice. Figure S3. The effects of YYFZBJS in intestinal tumorigenesis. Figure S4. The effects of YYFZBJS in intestinal tumor numbers. Figure S5. Heatmap of inflammatory cytokines analyses between C57BL/6 J mice and ApcMin/+ mice. Figure S6. The phenotype of IL-17-producing T cells Th17 in the spleen of ApcMin/+ mice was examined. Figure S7. The effect of YYFZBJS on CRC cell proliferation. [file 12964_2020_596_MOESM2_ESM.doc]

**Supplementary Data**

***Cell viability assays***

Cell proliferation was determined using the CCK-8 cell counting kit (Sigma-Aldrich). Briefly, cells were seeded in 96-well plates at 1×10^4^ cells/well as previously described (Sui *et al*., 2016). The CCK-8 assay was performed 48 h after treatment. Treated cells were incubated 4 h with culture medium containing the CCK-8 reagent, absorbance was recorded at 450 nm using a microplate enzyme-linked immunosorbent assay reader (Labsystems Dragon, Wellscan). The relative inhibition rate of cell growth was calculated according to the formula R
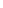
=
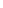
(A2-A1)/A2×100% and P
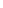
=
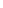
A1/A2×100%, in which R is the relative inhibition rate, P is the relative proliferation ratio for cell growth, A1 is the mean absorbance value of transfected cells, and A2 is the mean absorbance value of untransfected control cells without any drug treatment. All experiments were conducted using 5 wells per experiment and repeated at least three times.

***In vivo BrdU assay***

According to 5-bromo-2-deoxyuridine (BrdU) in vivo kit’s instructions(BrdU; Sigma-Aldrich), prepare a fresh BrdU solution at 10 mg/ml in saline every time and keep it refrigerated in the dark. The solution is sonicated in an ultrasound water bath for a few minutes immediately before injection. It was injected intraperitoneally into the mice once daily for 5 days as described previously[1-3], then intestine tissues was harvested after cleanout with PBS. The tissues were fixed on glass slides with 2.5% paraformaldehyde, Epitopes were retrieved by heat induction with Antigen Decloaker 10X (Biocare Medical, Concord, CA) in a rice cooker for 10 minutes at 120°C. After blocking non-specific binding (Protein Block, 30 minutes, room temperature), tissues were incubated for 2 hrs at RT (room temperature) with mouse anti-BrdU (1:100, BD Biosciences, San Jose, CA). Then the tissues were labeled for 1 hr at RT with AlexaFluor-488 goat anti-mouse IgG (1:200), AlexaFluor-647 goat anti-chicken IgG (1:200). The cell nuclei were stained twice for 10 minutes at RT with DAPI as a counter stain. Images were takenby the Leica DMi8 Laser Scanning Confocal.

***Participants***

Healthy controls and YYFZBJS users were recruited from asymptomatic volunteers who underwent colonoscopy with no significant abnormalities. All Subjects information was provided in the **Supplementary Table 4**. 10 volunteers took YYFZBJS by oral administration (51 mg, once per day) for 4 weeks. One gram of the mixed feces was diluted in 5mL sterile PBS solution, then initial filtered, concen-trated, homogenized, step by step filtered, centrifuged. The super-natant was collected, isolated, purified and equally repackaged before kept in −80°C refrigerator.

Both the group of volunteers taking YYFZBJS and the group of healthy donors have undergone rigorous screening and underwent informed consent for stool donation. The human study conformed “International ethical guidelines for biomedical research involving human subjects (2002)” developed by Council For International Organizations Of Medical Sciences (CIOMS) in collaboration with World Health Organization (WHO), which was approved by the ethics committee of Shuguang Hospital of Shanghai University of Traditional Chinese Medicine.

**Supplementary Table**

**Table 1. PCR primers**

| **gene** | **Forward primer** | **Reverse primer** |
| --- | --- | --- |
| Gata3 | TCCGGCTTCATCCTCTTCTCTGG | CTGTGCTGGATCGTGCCTTGG |
| T-bet | GTCGCTTCCTTGGATCCTTCGC | ACATTCGCCGTCCTTGCTTAGTG |
| TNF-α | GCGACGTGGAACTGGCAGAAG | CATCGGCTGGCACCACTAGTTG |
| FOXP3 | TCCAGGACAGACCACACTTCATGC | CTGGCTCCTCGAAGACCTTCTCAC |
| Ror-γt | ACAGCAGCAGCAACAGGAAC | GCCTCGTTCTGGACTATACTCAAG |
| APC | TTCCACTTTGGCATAAGGC | TTCTGAGAAAGACAGAAGTTA |
| c-Myc | TGACCTAACTCGAGGAGGAGCTGGAATC | AAGTTTGAGGCAGTTAAAATTATGGCTGAAGC |
| Axin2 | ATGCTAGGCGGAATGAAGATG | GGAGACAACGCTGTTGTTCTC |
| EphB3 | AAGAGACTCTCATGGACACGAAAT | ACTTCCCGCCGCCAGATG |
| β-catenin | ATGGGTAGGGCAAATCAGTAAGAGGT | AAGCATCGTATCACAGCAGGTTAC |
| TCF4 | CGAGTGCACGTTGAAAGAAA | ATGTGAAGCTGTCGCTCCTT |
| LEF1 | AGACATCCTCCAGCTCCTGA | GATGGATAGGGTTGCCTGAA |
| CyclinD1 | AGCTCCTGTGCTGCGAAG TGGAAA | AGTGTTCAATGAAATCGTG CGGGGT |
| Lgr5 | GAGGAAGCGCTACAGAATTTGAGA | GTGGCACGTAGCTGATGTGG |
| GAPDH | TGTGTCCGTCGTGGATCTGA | CCTGCTTCACCACCTTCTTGA |

**Table 2. Histopathologic analysis of neoplastic lesions and the degree of dysplasia**

| **Group** | **carcinoma/mouse number** | **malignant degree of carcinoma** |
| --- | --- | --- |
| Control | 8/8 | 100% |
| low-YYFZBJS | 3/8 | 37.5% |
| middle-YYFZBJS | 2/8 | 25% |
| high-YYFZBJS | 0/8 | 0% |
| Aspirin | 0/8 | 0% |

Histopathologic analysis of neoplastic lesions and the degree of dysplasia were assessed according to standard criteria and classification of adenomas of the colon. tubular adenoma with high-grade dysplasia characterized; low grade adenocarcinomas with focal submucosal invasion [4].

**Table 4. Clinical characteristics of the human donors for stool gavage to mice**

| Group | Age  (years) | Sex | BMI  (kg/m^2^) | Site of tumor | Family history of APC | Smoking | Drinking | Dietary  preference |
| --- | --- | --- | --- | --- | --- | --- | --- | --- |
| Healthy control | 54 | Male | 23.15 | N | N | Y | N | Normal diet |
|  | 46 | Male | 20.05 | N | N | N | N | Normal diet |
|  | 58 | Female | 21.93 | N | N | N | N | Normal diet |
|  | 33 | Female | 23.15 | N | N | N | N | Normal diet |
|  | 62 | Female | 23.74 | N | N | N | N | Sweet diet |
|  | 31 | Female | 19.49 | N | N | N | N | Spicy diet |
|  | 64 | Male | 22.60 | N | N | N | Y | Normal diet |
|  | 44 | Female | 23.53 | N | N | N | N | Normal diet |
|  | 37 | Male | 22.10 | N | N | N | N | Normal diet |
|  | 54 | Male | 20.76 | N | N | Y | N | Normal diet |
| Healthy cases  With YYFZBJS | 59 | Female | 23.01 | N | N | N | N | Normal diet |
|  | 33 | Female | 20.70 | N | N | N | N | Spicy diet |
|  | 43 | Male | 20.76 | N | N | N | N | Normal diet |
|  | 59 | Female | 21.80 | N | N | N | N | Normal diet |
|  | 64 | Male | 23.55 | N | N | Y | N | Normal diet |
|  | 71 | Male | 23.36 | N | N | N | N | Normal diet |
|  | 47 | Male | 23.67 | N | N | N | N | Sweet diet |
|  | 62 | Male | 20.98 | N | N | N | N | Normal diet |
|  | 35 | Male | 21.20 | N | N | N | Y | Normal diet |
|  | 55 | Female | 20.54 | N | N | N | N | Sweet diet |

YYFZBJS: YiYi Fuzi Baijiang San,BMI:Body Mass Index;Y:Yes;N:No

**Supplementary Figure**

**
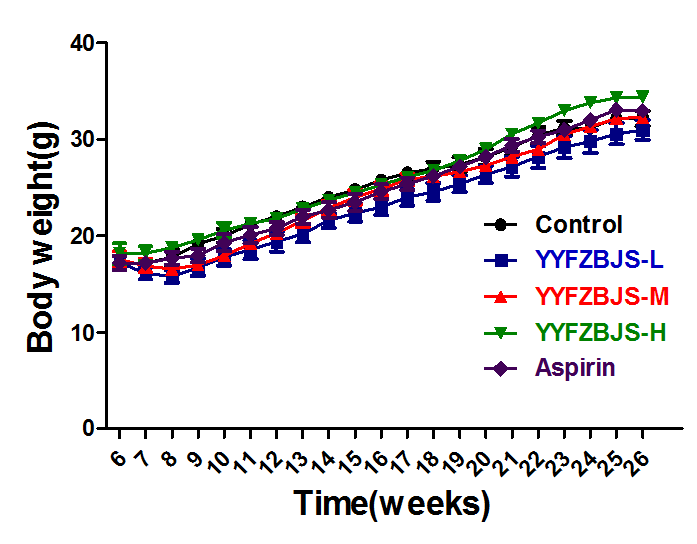
**

**Supplementary Figure 1 The effect of YYFZBJS on body weights of *Apc^Min/+^* mice**

Body weights of ApcMin/+ mice that were orally treatment with YYFZBJS for 20 weeks.


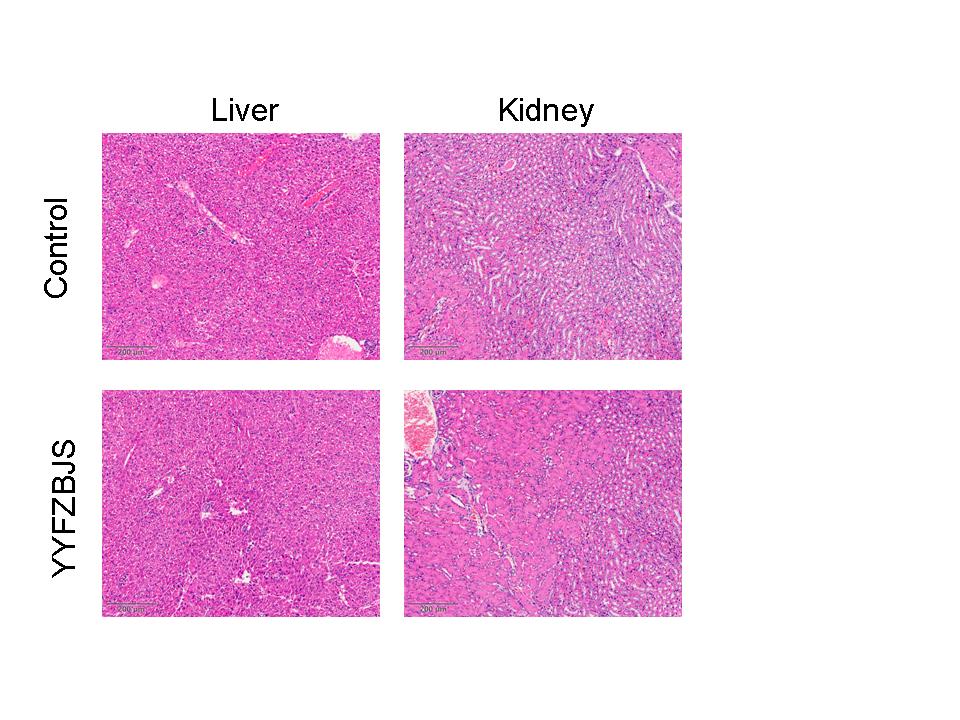


**Supplementary Figure 2 The effects of YYFZBJS on the liver and kidney in *Apc^Min/+^* mice**

The mice are from the model of *Apc^Min/+^* mice. Liver and kidney were stained with HE and observed under phase-contrast microscope. Scale bars, 200 μm.


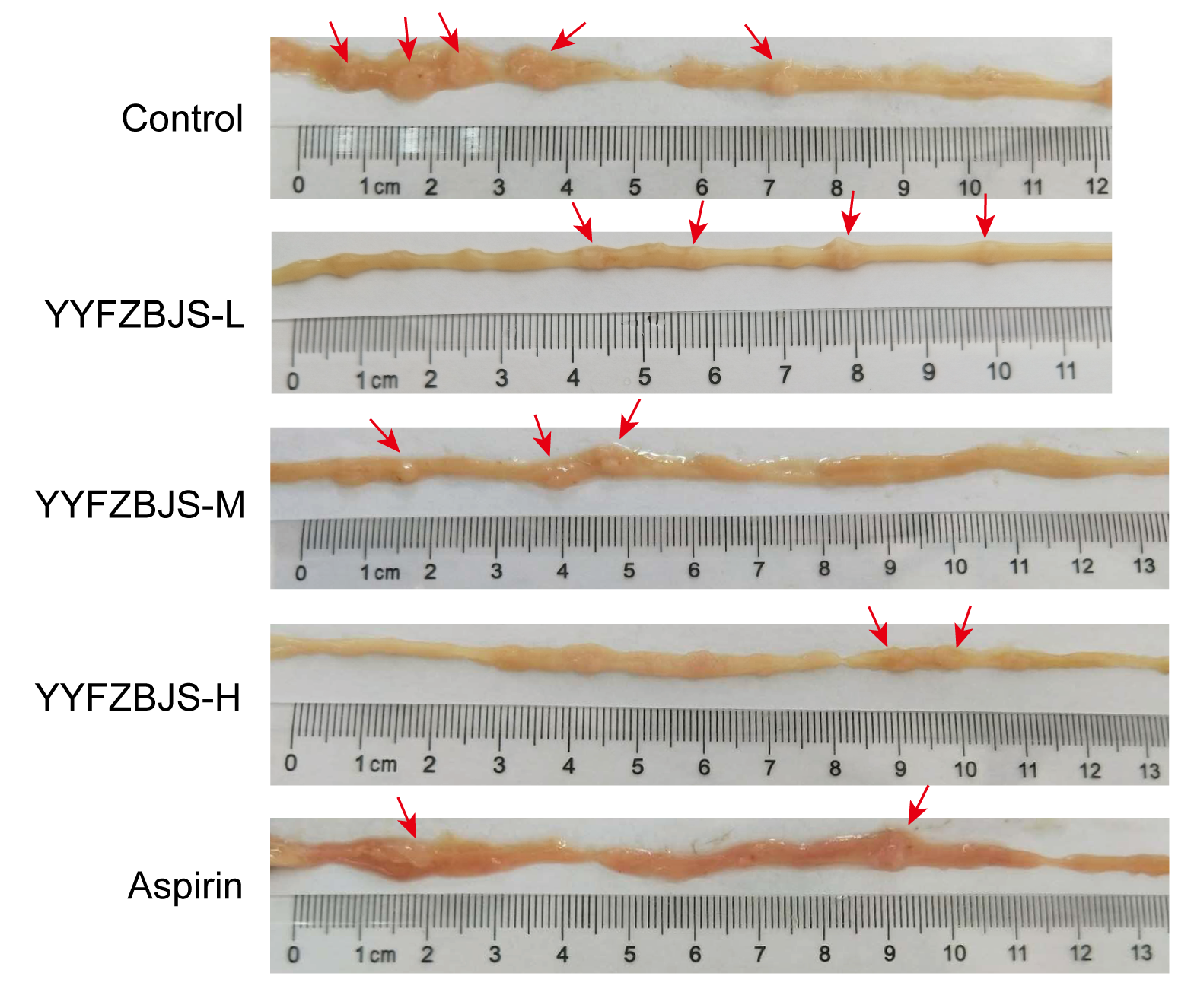


**Supplementary Figure 3 The effects of YYFZBJS in intestinal tumorigenesis**

Macroscopic view of the representative mouse intestinal shows several polypoid and discoid colonic tumors from different groups of *Apc^Min/+^* mice.


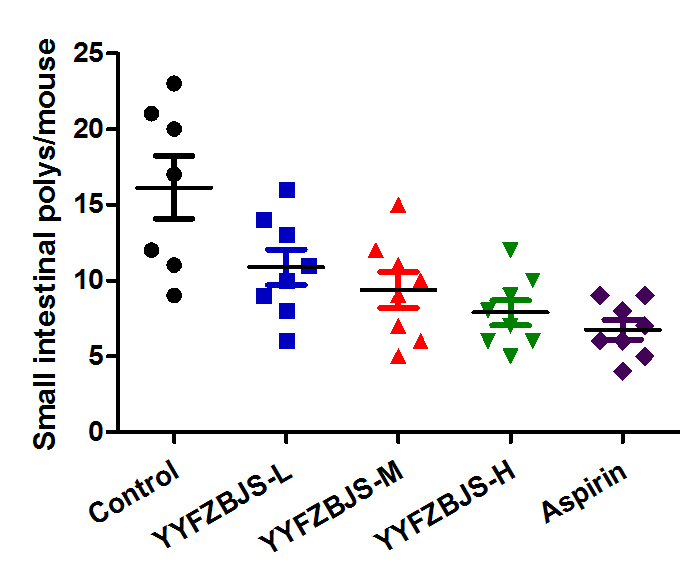

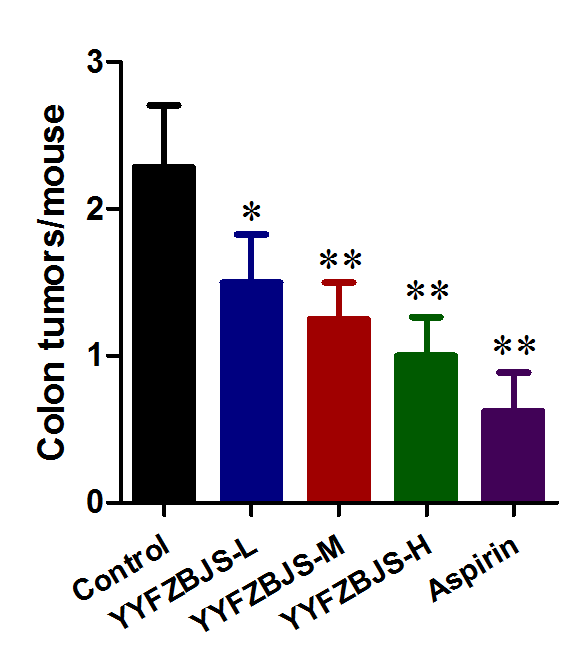

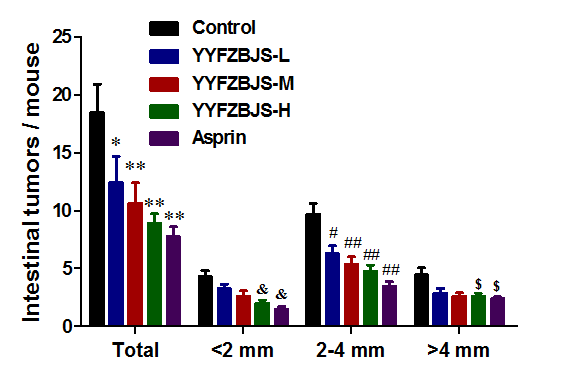


**Supplementary Figure 4 The effects of YYFZBJS in intestinal tumor numbers**

(A) The number of intestinal polyps in small intestinal from different groups of *Apc^Min/+^* mice. (B) The number of intestinal polyps in the colon from different groups of *Apc^Min/+^* mice. (C) The tumor size distribution in the intestine was listed and compared with control. The data are presented as the mean ± SD from at least three experiments.


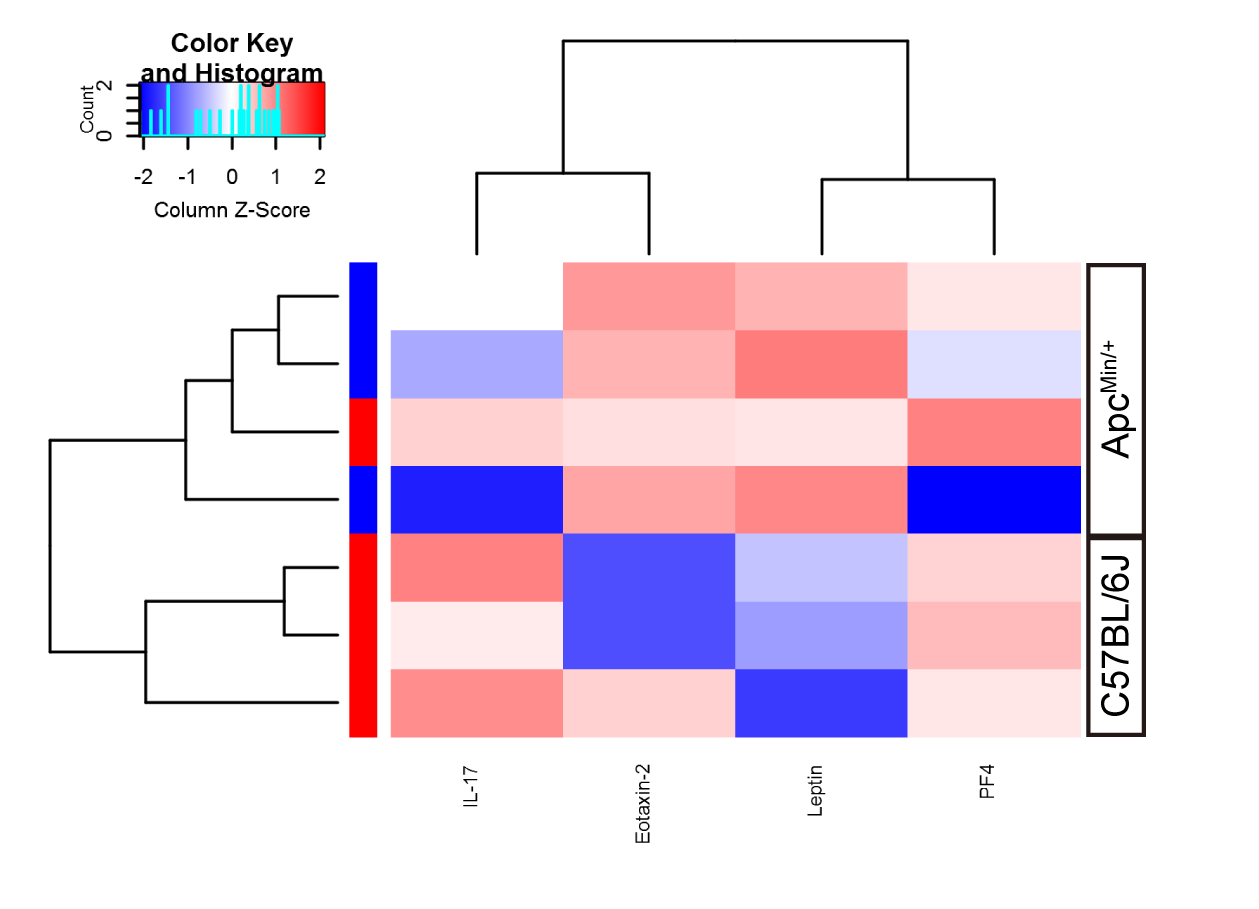


**Supplementary Figure 5 Heatmap of inflammatory cytokines analyses between C57BL/6J mice and *Apc^Min/+^* mice**

4 differentially expressed factors have been analyses by the cytokine antibody array (Ray Biotech)[5].

**
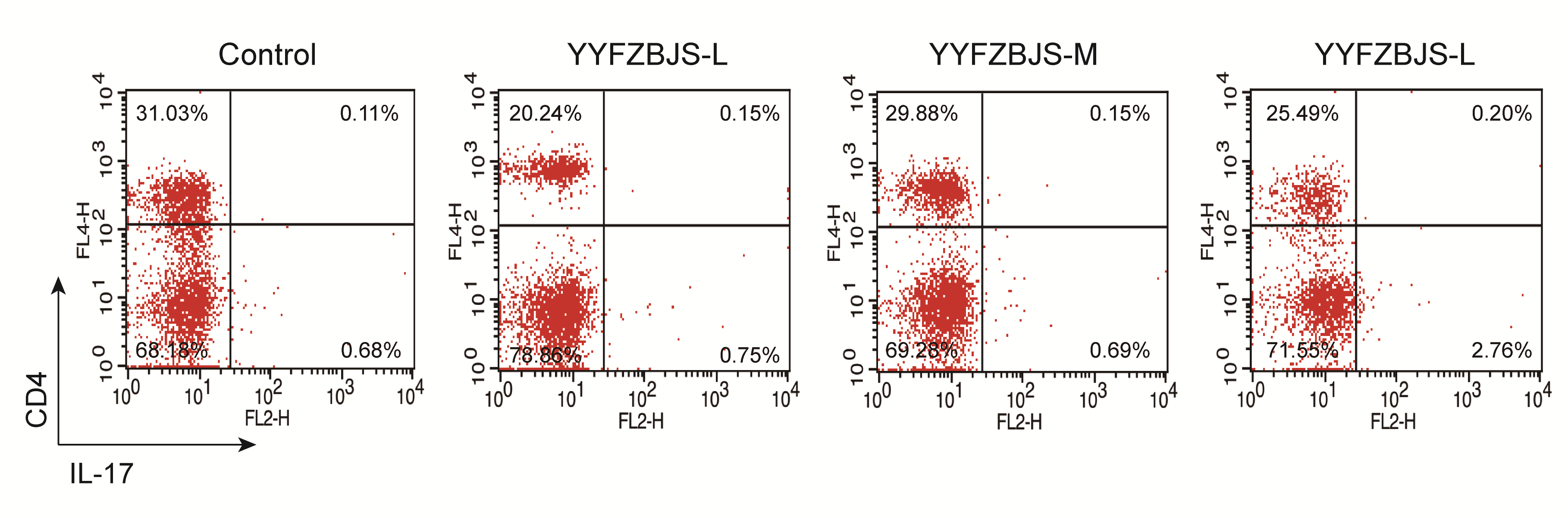
**

**Supplementary Figure 6 The phenotype of IL-17-producing T cells Th17 in the spleen of *Apc^Min/+^* mice was examined.**

Representative flow cytometry plots of spleen lymphocyte showing the viable lymphocyte gate on CD4+, IL-17+ T cells.

**
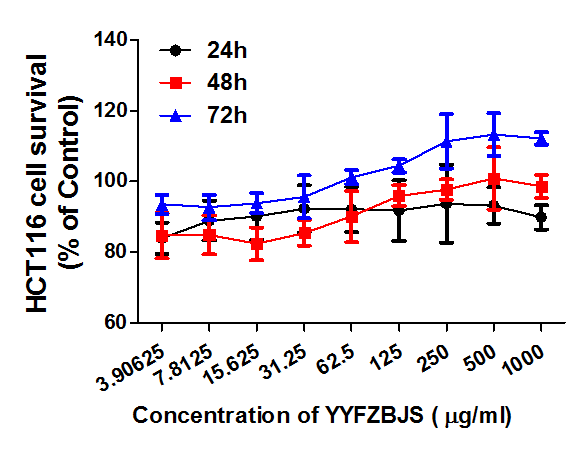

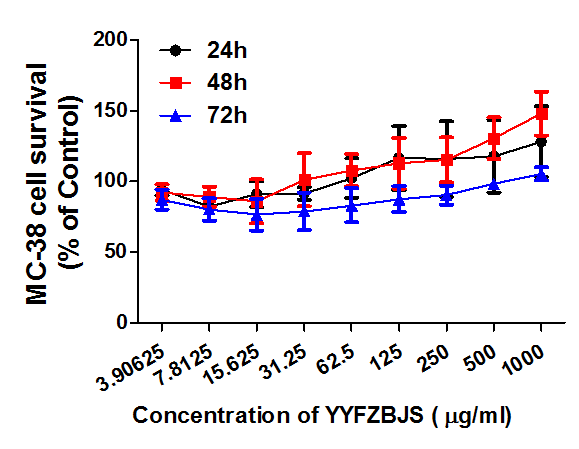
**

**Supplementary Figure 7 The effect of YYFZBJS on CRC cell proliferation.**

Cells were treated with various concentrations of YYFZBJS for 24, 48 and 72 h and analysis by CCK-8 analyses.

**Reference**

1. Ting Fu, Sally Coulter, Eiji Yoshihara,Tae Gyu Oh, Sungsoon Fang, Fritz Cayabyab，et al. FXR Regulates Intestinal Cancer Stem Cell Proliferation. Cell. 2019;176:1098-112.
2. A.V. Capuco. Identification of putative bovine mammary epithelial stem cells by their retention of labeled DNA strands.Exp. Biol. Med. 2007;232:1381-90.
3. Toshiyuki Yamamoto, Atsuyoshi Mita, Camillo Ricordia, Shari Messinger, Atsushi Miki, Yasunaru Sakuma,et al. Prolactin Supplementation to Culture Medium Improves Beta Cell Survival.Transplantation. 2010;89(11):1328-35.
4. Poutahidis T, Rao VP, Olipitz W, Taylor CL, Jackson EA, Levkovich T, Lee CW, Fox JG, Ge Z, Erdman SE. CD4+ lymphocytes modulate prostate cancer progression in mice. Int J Cancer. 2009;125(4):868-78.
5. Lee, HJ, Zhuang, G, Cao, Y, Du, P, Kim, HJ, Settleman, J. Drug Resistance via Feedback Activation of Stat3 in Oncogene-Addicted Cancer Cells. Cancer Cell. 2014;26:207-21.
